# Single-cell analysis of lung adenocarcinoma cell lines reveals diverse expression patterns of individual cells invoked by a molecular target drug treatment

**DOI:** 10.1186/s13059-015-0636-y

**Published:** 2015-04-03

**Authors:** Ayako Suzuki, Koutatsu Matsushima, Hideki Makinoshima, Sumio Sugano, Takashi Kohno, Katsuya Tsuchihara, Yutaka Suzuki

**Affiliations:** Department of Medical Genome Sciences, Graduate School of Frontier Sciences, The University of Tokyo, Chiba, 277-8562 Japan; Division of TR, The Exploratory Oncology Research and Clinical Trial Center, National Cancer Center, Chiba, 277-8577 Japan; Division of Genome Biology, National Cancer Center Research Institute, Tokyo, 104-0045 Japan; Division of TR, The Exploratory Oncology Research and Clinical Trial Center, National Cancer Center, Tokyo, 104-0045 Japan; Department of Computational Biology, Graduate School of Frontier Sciences, The University of Tokyo, Chiba, 277-8562 Japan

## Abstract

**Background:**

To understand the heterogeneous behaviors of individual cancer cells, it is essential to investigate gene expression levels as well as their divergence between different individual cells. Recent advances in next-generation sequencing-related technologies have enabled us to conduct a single-cell RNA-Seq analysis of a series of lung adenocarcinoma cell lines.

**Results:**

We analyze a total of 336 single-cell RNA-Seq libraries from seven cell lines. The results are highly robust regarding both average expression levels and the relative gene expression differences between individual cells. Gene expression diversity is characteristic depending on genes and pathways. Analyses of individual cells treated with the multi-tyrosine kinase inhibitor vandetanib reveal that, while the ribosomal genes and many other so-called house-keeping genes reduce their relative expression diversity during the drug treatment, the genes that are directly targeted by vandetanib, the EGFR and RET genes, remain constant. Rigid transcriptional control of these genes may not allow plastic changes of their expression with the drug treatment or during the cellular acquisition of drug resistance. Additionally, we find that the gene expression patterns of cancer-related genes are sometimes more diverse than expected based on the founder cells. Furthermore, we find that this diversity is occasionally latent in a normal state and initially becomes apparent after the drug treatment.

**Conclusions:**

Characteristic patterns in gene expression divergence, which would not be revealed by transcriptome analysis of bulk cells, may also play important roles when cells acquire drug resistance, perhaps by providing a cellular reservoir for gene expression programs.

**Electronic supplementary material:**

The online version of this article (doi:10.1186/s13059-015-0636-y) contains supplementary material, which is available to authorized users.

## Background

Recent advances in next-generation sequencing analysis have enabled genome and transcriptome analysis of a large number of samples within a reasonable time and at a reasonable cost. Particularly, whole-genome sequencing and exome sequencing analyses have been conducted intensively to characterize somatic mutations in cancer. Recently, The Cancer Genome Atlas and the International Cancer Genome Consortium have reported genome, RNA and DNA methylation patterns for thousands of clinical samples for hundreds of diverse cancer types [1,2].

Advances in next-generation sequencing are not limited to the throughput and cost of sequencing itself. Technical innovations in the sample preparation steps have also significantly improved, enabling us to construct a sequencing library from a very small amount of starting material. For the purpose of genome sequencing, multiple displacement amplifications [3] are now widely used to amplify sub-picogram genomic DNA to prepare a sequencing template from a single cell [4]. Additionally, for the purpose of transcriptome analysis, several methods for whole transcriptome amplification, including template switching-based cDNA amplification, have been developed, enabling transcriptome analysis of a single cell [5,6]. Although it has been thought that amplification bias would introduce significant bias in the expression information during the amplification step, it is now possible to prepare an RNA-Seq library in a high-throughput and reasonably reproducible manner [7]. At the same time, methods to capture a single cell in a high-throughput manner are also being rapidly developed. Using microfluidics technology or cell sorters, commercial instruments now support automatic separation of cells, which are subsequently used for template preparation for sequencing analysis in a seamless manner [8]. Taken together, these methods have opened the possibility to conduct genome or transcriptome analysis of a single cell in various biological systems [9].

With the analytical methods for individual cells available, one of the most attractive objectives for their application should be single-cell analysis of cancer cells. The extent to which cancer cells are diverse within a given population and how they respond to environmental changes, particularly to an anti-cancer drug treatment, are pressing research questions. Indeed, these questions have been analyzed for a limited number of genes. For example, the single-cell transcriptome of colon cancer was described in a previous study, which reported the results of quantitative PCR for a limited number of cancer-related genes [10]. That study revealed that transcriptional diversity of cancer tissues should be explained by multilineage differentiation of the individual cancer cells and that such diversity is closely associated with prognostic outcomes. However, comprehensive knowledge of how individual cells change their transcriptional programs in response to environmental changes remains elusive.

In this study, we characterized the heterogeneity in gene expression that exists within a given population of cancer cells. We also attempted to investigate how the transcriptome of each cell responds to a molecularly targeted drug and how they differ between parental cells and cells that have acquired drug resistance. For this purpose, we used a series of lung adenocarcinoma-derived cell lines. We constructed single-cell RNA-Seq libraries and screened them for heterogeneous transcriptome features. We characterized distinct transcriptome features, separating individual cells in a particular cell type and those in different cell types. We put particular focus on the analysis of LC2/ad. This cell line expresses a fusion gene transcript of a tyrosine kinase, RET, and CCDC6, resulting in the aberrant activation of the kinase activity of RET, which serves as a major driving force for carcinogenesis (a cancer driver) [11,12]. Indeed, at the clinical level, the RET fusion transcripts were found in 1 to 2% of lung adenocarcinomas. A multi-tyrosine kinase inhibitor, vandetanib, which inhibits the tyrosine kinase activity of RET, is expected to be effective in treating patients expressing these fusion transcripts [13-16]. Actually, several 'proof of concept' clinical trials are ongoing. However, acquiring drug resistance to vandetanib will be unavoidable, as has occurred for other tyrosine kinase inhibitors, including gefitinib for EGFR and crizotinib for ALK. Indeed, we and others have identified a subclone of LC2/ad that has acquired resistance to vandetanib (LC2/ad-R; see below). In this study, we examined the gene expression patterns in individual cells of LC2/ad and LC2/ad-R cells with or without vandetanib treatment. Here, we describe our single-cell RNA-Seq analysis using 336 single-cell RNA-Seq libraries constructed from seven types of lung adenocarcinoma cell lines.

## Results and discussion

### RNA-Seq analysis of individual cells of a lung adenocarcinoma cell line, LC2/ad

**Figure 1:**
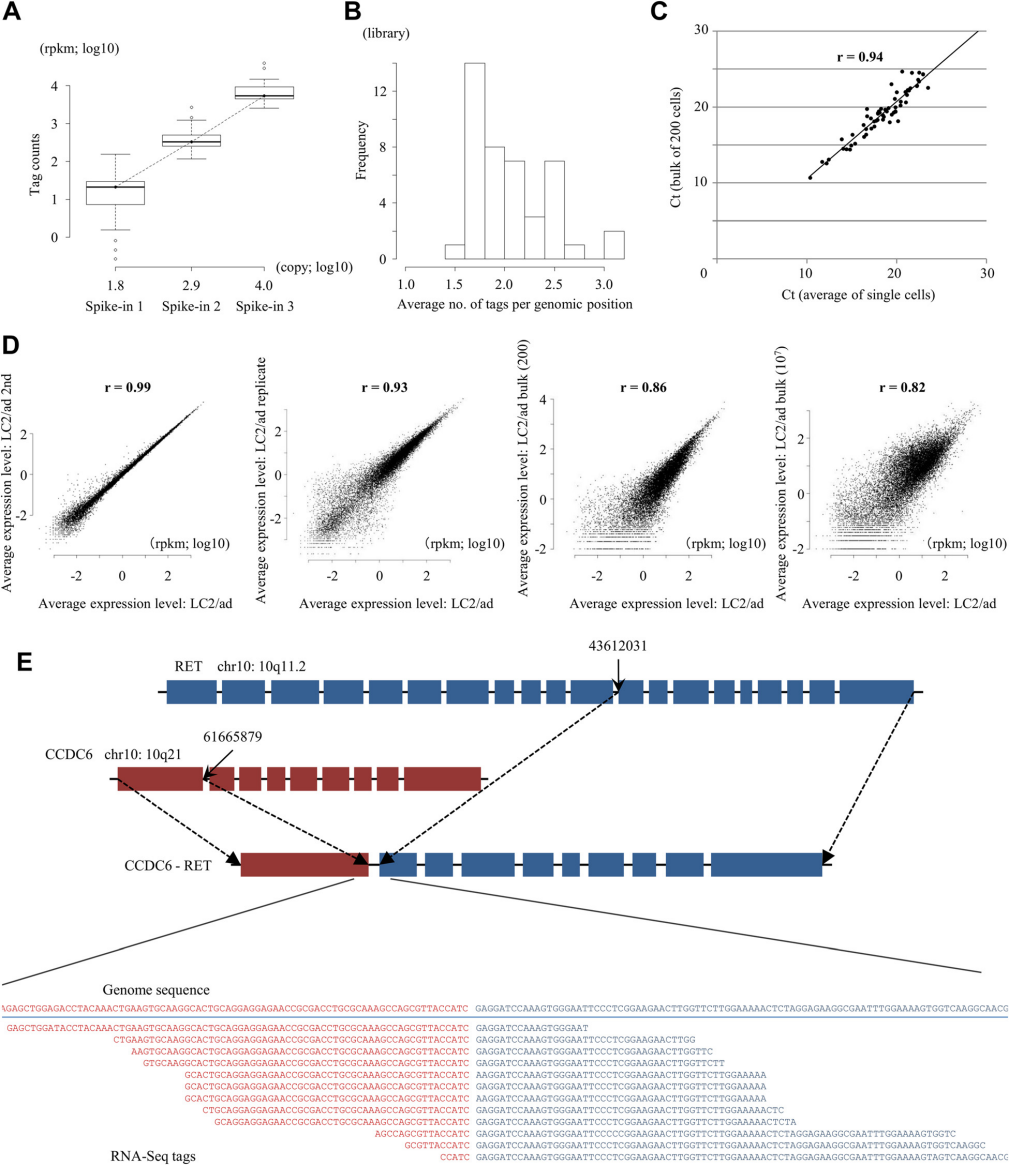
Generation of the RNA-Seq data from single cells of LC2/ad. **(A)** Read counts of spike-in controls. The tag counts corresponding to the indicated spike-ins are represented on the y-axis. The x-axis represents the copy numbers of the indicated spike-ins mixed in the sample. rpkm, reads per million tags per kilobase mRNA. **(B)** Complexity of the sequence reads. The number of RNA-Seq tags mapped to the same genomic position is shown. **(C)** Validation analysis using real-time PCR. Quantitative RT-PCR was conducted using first-strand cDNA for the genes listed in Additional file 3. Ct values were compared between the average of individual cells and those of the bulk of 200 cells. **(D)** Comparison between sequence duplicates (first panel), between biological duplicates (second panel) and between bulk and individual cells (third and fourth panels). The relation between gene expression levels measured from the average of independent cells and bulk RNA-Seq analysis of 200 cells (third panel) and >10^7^ cells (fourth panel) are shown. Pearson’s correlation between two experiments is shown in the plot. **(E)** Identification of the fusion gene transcript, CCDC6-RET, using the RNA-Seq tags of single cells. The number of tags that directly spanned the junction point of the gene fusion is shown. In the upper panel, the densities of the RNA-Seq tags that were mapped to the indicated genomic positions (the RET gene region in the right half and the CCDC6 gene region in the left half) are also shown (in blue and red letters, respectively). The results in LC2/ad cells are shown. Note that even in the case where there was no RNA-Seq tag directly spanning the junction point, the distribution of the RNA-Seq tags were significantly different between the 5’ and 3’ halves of the RET gene, which indicates the discontinuity of this transcript.

To analyze gene expression levels and their variances between different individual cells, we constructed a series of single-cell RNA-Seq libraries from a human lung adenocarcinoma cell line, LC2/ad. To construct the libraries, we used the Fluidigm C1 platform (for details on the procedure, see Figure S1 in Additional file 1) [8]. Using the constructed libraries, we generated RNA-Seq tags by 97-base paired-end reads. We allocated a full flow cell of HiSeq2500 with 12-plex samples to a single lane, yielding 14 million tags, on average, for each library (Additional file 2). For the purpose of the initial quality check, we utilized three spike-in controls. Most of the cells were within the range of standard deviations regarding the expected read counts for all of the spike-in controls (Figure 1A). To further ensure the fidelity of the data, we discarded libraries in which tag counts of any of the spike-in controls deviated by more than two standard deviations from the other cells. Forty-three libraries passed the filter and were used for the following analyses (Table 1). RNA-Seq tags derived from these libraries were mapped to the reference human genome allowing two base mismatches. Among the mapped RNA-Seq tags, an average of 78% were mapped within the RefSeq gene regions, which is comparable with normal RNA-Seq libraries. To measure the gene expression levels, we counted the RNA-Seq tags that were mapped to the RefSeq regions and calculated reads per million tags per kilobase mRNA (rpkm) [17]. Further details of the statistics are shown in Additional file 2.

**Table 1:** **Statistics of the RNA-Seq tag data used for the present study**

	**Number of libraries**	**Average mapped tags**	**Average mapped in RefSeq regions**	**Average complexity**
LC2/ad	43	4,567,666	3,581,044 (78%)	2.3
LC2/ad (replicate)	45	8,909,696	7,190,460 (81%)	2.6
LC2/ad-R	70	9,456,920	7,052,916 (75%)	3.7
LC2/ad + van	28	7,949,208	6,408,497 (81%)	2.3
LC2/ad-R + van	58	4,324,350	2,926,954 (68%)	2.7
PC-9	46	7,409,611	5,726,548 (77%)	2.4
VMRC-LCD	46	6,825,661	5,059,441 (74%)	2.5

For the obtained results, we conducted a series of validation analyses. First, to estimate potential PCR duplicates in the RNA-Seq tags, we counted the frequency of the tags that had identical sequences (giving the same start- and end-mapping coordinates). We found that, on average, such tags appeared 2.6 times per genomic position (Figure 1B), which is almost at a similar rate as usual RNA-Seq libraries at this depth (Table S2 in Additional file 1). Second, to validate equal amplification of cDNAs between different cells, we performed quantitative RT-PCR analysis of 85 genes (Additional file 3). As shown in Figure 1C, the quantitative RT-PCR results were well-correlated (r = 0.94) between RNA-Seq tags from a bulk library of 200 cells and an average of 43 single cell libraries, although this experiment did not directly support equal amplification between different cells. Third, we examined the reproducibility of the data. We repeated the sequencing using the same templates and found that the correlation was almost perfect (r = 0.99; the first panel in Figure 1D). We also analyzed and found that the results are robust for the increasing sequence depth and the re-amplification of the same single cell materials (Figure S2 in Additional file 1). To further ensure the reproducibility between independent experiments, we repeated the library construction, starting from independently cultured LC2/ad cells. Again, we found that the results were highly reproducible (r = 0.93; the second panel in Figure 1D). To examine reproducibility with regard to dependence on the number of starting cells and the library construction protocol, we compared the results of the single-cell analysis with those obtained from the libraries prepared from 200 cells and those from the libraries constructed according to the usual RNA-Seq protocol using 10 million cells. We observed reasonable reproducibility with r = 0.86 and r = 0.82 (the third and fourth panels in Figure 1D). Last, we examined whether the characteristic fusion gene transcript CCDC6-RET can be detected in the single-cell libraries. As shown in Figure 1E, we searched and identified a total of 12 RNA-Seq tags that spanned the junctions of the fusion gene (also see Figure S3 in Additional file 1 for identification of the tags of the fusion transcript from the increased sequence depth; identification of the tags spanning the driver mutation in the EGFR gene in a different cell line, PC-9, is also described there). Taken together, these results demonstrate that the single-cell data should be reproducible and can be used similarly to usual RNA-Seq analyses.

### Gene expression divergence between different individual cells

**Figure 2:**
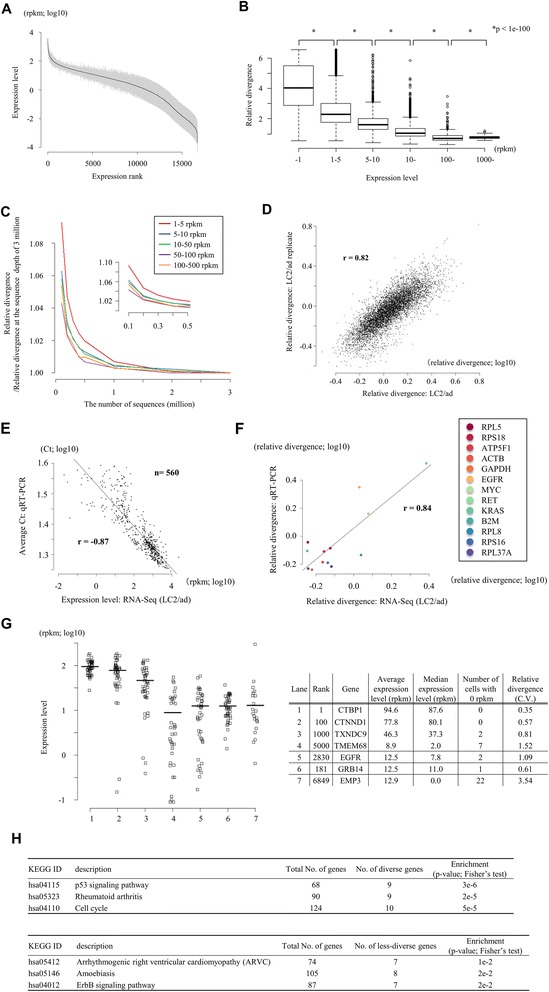
Diversity in the expression levels between different individual cells and different genes. **(A)** Distribution of the average gene expression levels (solid line) and the relative standard deviations (vertical lines). **(B)** Relation between average expression levels and the relative divergence. Statistical significance calculated by Fisher’s exact test (f-test) is shown in the margin. **(C)** Dependency of the calculated relative divergence on the varying sequence depth per cell. Average values for the indicated populations are shown. A total of 2,370, 1,014, 3,489, 541 and 429 genes were used for genes with average expression levels of 1 to 5, 5 to 10, 10 to 50, 50 to 100, and 100 to 500 rpkm, respectively. The inset represents magnification of the main plot at the region of small values on the x-axis. **(D)** Reproducibility of the experiments with regard to expression variation. Relative expression variation obtained from two independent experiments is shown. Pearson’s correlation is shown in the plot. **(E,F)** Validation analysis using real time RT-PCR assays in individual cells of LC2/ad. A total of 13 genes were analyzed. Pearson’s correlation coefficients are shown in the plot. (E) Relation between Ct values of real time RT-PCR assays and the results of single-cell RNA-Seq analyses. Each dot represents the average of triplicate experiments. (F) Relation between the relative divergence calculated based on the real time RT-PCR analyses and the single-cell RNA-Seq analyses. **(G)** Relative divergence in different genes. Genes were sorted according to their relative divergence and genes that were ranked at 1, 10, 100, 1,000 and 5,000 are shown. The horizontal bar represents the average expression level. The EGFR gene, which was ranked 2830, is also shown. CV, coefficient of variation. **(H)** Gene Ontology terms (upper panel) and KEGG (Kyoto Encyclopedia of Genes and Genomes) pathways (lower panel) for genes that showed highly diverse expression between individual cells.

Using the generated RNA-Seq data, we first examined the gene expression levels averaged for the individual cells. As previously reported, expression levels showed a distribution that roughly follows Zipf’s law (bold line in Figure 2A) [18]. In addition to the average expression levels, we also investigated divergence of the expression levels among the individual cells (pale vertical lines in Figure 2A). We calculated the standard deviation of the rpkm for each gene and divided it by the average rpkm (called 'relative divergence' hereafter). We found that a gene with a higher expression level tends to have a lower relative divergence (Figure 2B). This may have been caused by insufficient coverage of RNA-Seq tags, particularly for lowly expressed genes. However, at least for the genes with >5 rpkm, they were represented by approximately >50 RNA-Seq tags at this sequence depth; thus, the depth seemed to be sufficient to represent the truly divergent gene expression in cells (see Figure S5, S6 and S7 in Additional file 1 for further detailed analysis on the dependency of the sequence depth and the detected relative divergence; also see the relation between the sequence depth and the number of tags in each gene in Figure S8 in Additional file 1).

To further ensure the correct measurement of the relative divergence depending on the sequence coverage, we examined the dependency of the calculated relative divergence on varying sequencing depth. As shown in Figure 2C, calculated relative divergence plateaued when the sequence depth exceeded two million tags per cell, especially for genes with expression levels >5 rpkm. Additionally, it was unlikely that the observed divergences were derived from typical technical errors because they were reproducible between the independent experiments (r = 0.82; Figure 2D). Lastly, we conducted real time RT-PCR assays for 13 genes for each of the single cells (total data point n = 560) using the remaining aliquots of the amplified cDNAs (Figure 2E,F; see Table S4 in Additional file 1 for primers). We confirmed that, generally, the expression levels detected by real time RT-PCR were consistent with the results of RNA-Seq. We further compared the relative divergence between that calculated from the real time RT-PCR and that from the RNA-Seq and found that they are reasonably consistent (r = 0.84; also see Figure S4 in Additional file 1 for the case of PC-9, where r = 0.92 when calculated using total data points n = 630). Also, we examined the dependency of the observed relative divergence on the number of the cells used for the analysis. For this purpose, we used the dataset of LC2/ad cells (a total of 88 cells; LC2/ad (43 cells) + LC2/ad replicate (45 cells)). As shown in Figure S9 in Additional file 1, we found approximately 30 cells should be the minimum number of cells to estimate the relative divergence in gene expression, although even with 88 cells the plots did not seem to always reach a complete plateau. Based on these results, we concluded that the observed diversities in gene expressions were not derived merely from technical errors or insufficient sequence depth or inadequate data size but represent real biological phenomena.

We found that the divergences were frequently unique to genes, even between genes with similar expression levels (see below). For example, the relative divergences of the GRB14, EMP3 and EGFR genes (relative divergence ranks of 181, 6,849 and 2,830, respectively) were significantly different in spite of their similar average expression levels (Figure 2G). In this study, we evaluated the diversity in gene expression by considering their standard deviations. It should be noted, however, that the 'relative divergence' may not correctly reflect the overall divergence, particularly when there is a certain population of individual cells giving extremely high or low expression values. Indeed, the high relative divergence observed for EMP3 gene expression was accounted for by the fact that the expression of this gene was 0 in about half of the cells.

Then, we examined how many genes had diverse or less diverse expression patterns compared with genes with similar expression levels because the relative divergence showed some dependency on the average gene expression level. For this purpose, we matched the expression levels of the control genes: they were selected so that their average expression levels fell within two-fold of the expression levels of the original genes (though their relative divergence may vary). We selected 1,000 genes (or all of the genes satisfying the criterion) for each gene and selected the genes giving *P* < 0.05 (f-test) compared with the background distribution of the relative divergence at similar expression levels in the tests (see Materials and methods). We found a total of 305 and 596 genes that showed more or less diverse expression patterns, respectively (see Additional file 4 for a full list). Next, we examined whether the genes with high or low relative divergence were enriched in particular pathways. Genes of characteristic KEGG (Kyoto Encyclopedia of Genes and Genomes) pathways [19] were identified. In particular, those pathways related to cancers, such as the p53 signaling pathway (KEGG pathway ID: hsa04115; average *P* = 3e-6) and the ErbB signaling pathway (KEGG pathway ID: hsa04012; average *P* = 2e-2), were identified as pathways in which genes with expression patterns that had high and low diversity, respectively, were enriched (Figure 2H). Relative divergence may reflect the intrinsic properties of transcriptional regulatory machineries, which are characteristic depending on genes and pathways.

### Gene expression patterns in different lung adenocarcinoma cell lines

To characterize relative divergences in different cell lines, we conducted a similar single-cell RNA-Seq analysis for two additional lung adenocarcinoma cell lines, PC-9 and VMRC-LCD (Table 1). At the same time, we conducted whole-genome sequencing for LC2/ad, PC-9 and VMRC-LCD as bulk samples [20] (Figure S10 in Additional file 1). Consistent with previous studies, we detected the RET fusion gene as a driver mutation in LC2/ad (Figure S10B in Additional file 1). We also found PC-9 carries a known driver mutation in the EGFR gene (E746_A750del; Figure S10C in Additional file 1), while no driver was identified for VMRC-LCD. Similar to the case of LC2/ad, validation analyses of the RNA-Seq are shown in Figure S11 in Additional file 1.

**Figure 3:**
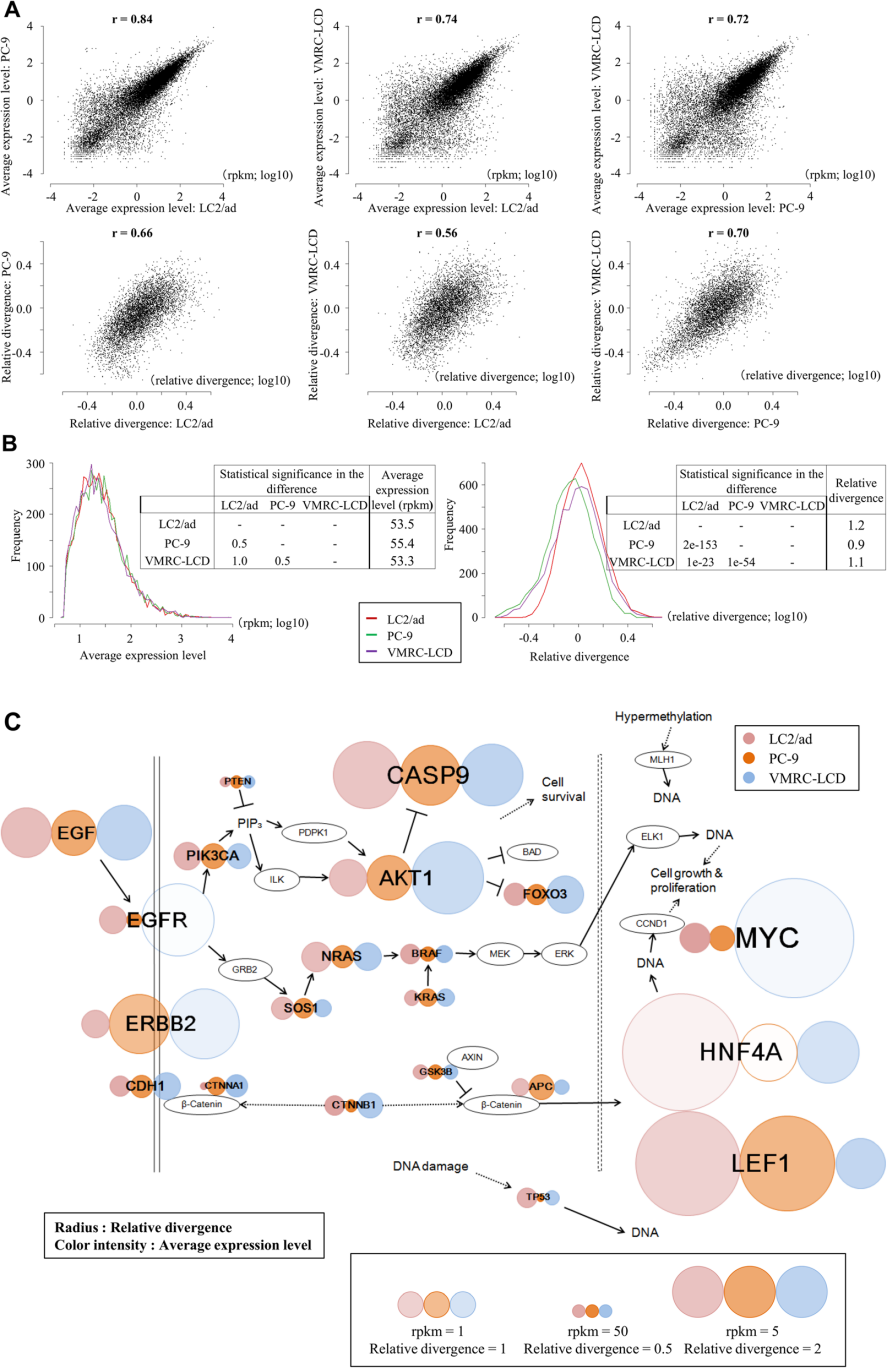
Expression diversity in different cell types. **(A)** Difference in the average gene expression levels (upper panels) and the relative divergences (lower panels) between LC2/ad and PC-9 cells (left panels), LC2/ad and VMRC-LCD cells (middle panels) and PC-9 and VMRC-LCD cells (right panels). Pearson’s correlation co-efficient is also shown in the plots. **(B)** Range of the average expression levels (left panel) and their relative divergences (right panel) in the indicated cell types (LC2/ad, red; PC-9, green; VMRC-LCD, purple). Statistical significance of the difference between the indicated cell lines and the average values are shown in the insets. **(C)** Expression pattern of the EGFR pathway genes. The color density of each circle represents the average expression level and the radius the relative deviation. Expression patterns of the indicated cell types are shown.

Using the obtained data, we compared the gene expression levels averaged for different individual cells. We found that they are generally similar between the cell lines, perhaps reflecting the fact that they were all established from lung adenocarcinomas (upper panels in Figure 3A). We also examined the distribution of the average gene expression levels. Again, we found that they are similar between cell lines (left panel in Figure 3B). However, the relative divergences were more distinct between cell types (lower panels in Figure 3A). For example, PC-9 gave the smallest expression variation between individual cells, followed by VMRC-LCD and LC2/ad (right panel in Figure 3B). Those differences were statistically significant (insets in Figure 3B).

As for LC2/ad, we conducted KEGG pathway enrichment analysis of the genes with high relative divergences in these cell lines. Again, we found that cancer-related genes and pathways were enriched (Table S6 in Additional file 1). However, we also found that each cell type had partly common and partly unique enrichment patterns (see below). Notably, we compared the gene expression patterns of the EGFR pathway genes, which are supposed to play pivotal roles in carcinogenesis of lung cancers [19,21], between cell lines (Figure 3C). We found that this pathway was, indeed, enriched for diversely or less diversely expressed genes in general, but different gene components contributed to the overall high divergence depending on the cell line (Figure 3C; Additional file 5). These divergences may contribute to the characteristic cellular behavior of each cell line in addition to their average expression levels.

### Gene expression patterns in cancer-related genes

**Figure 4:**
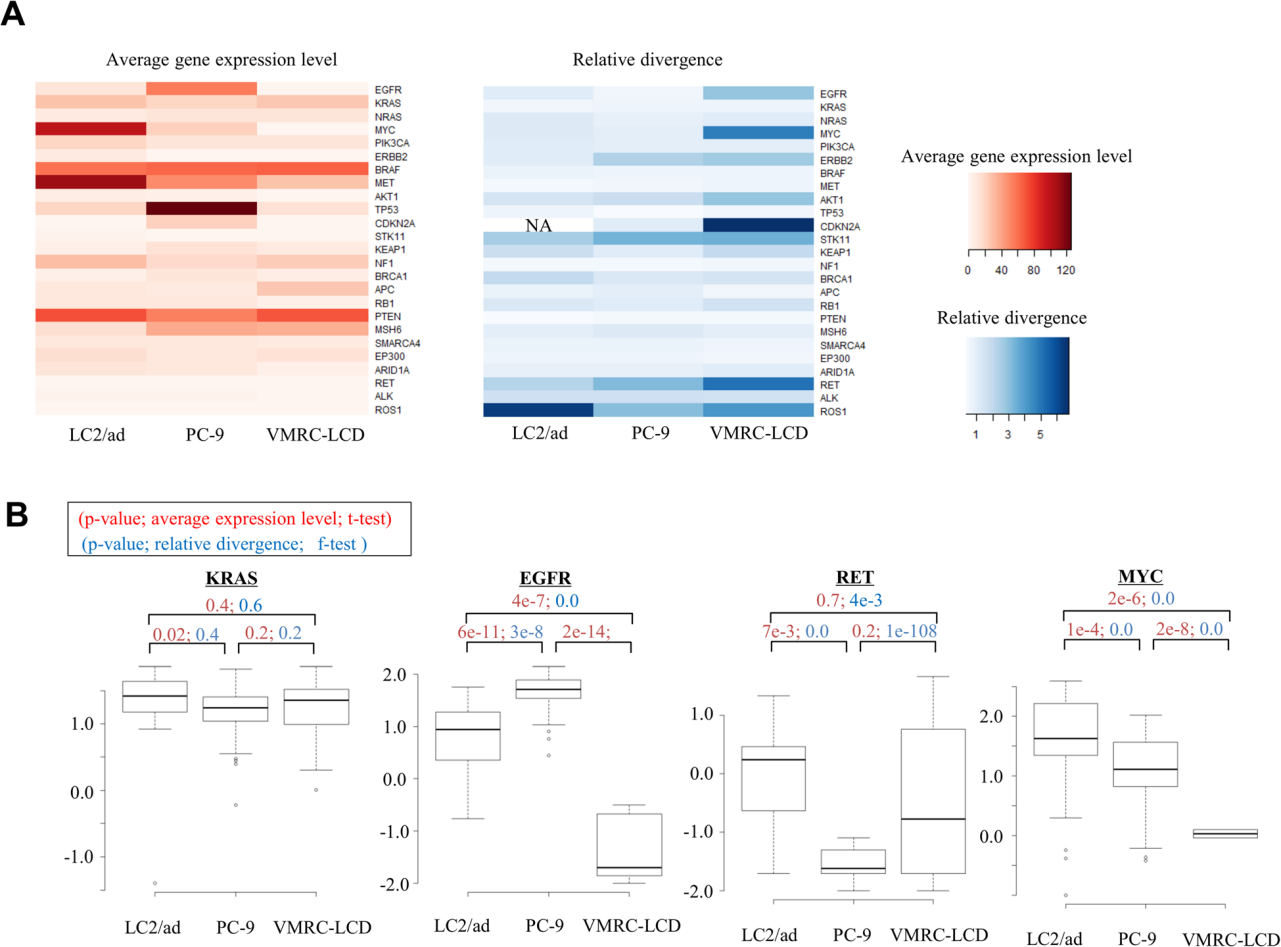
Expression diversity in the cancer-related genes. **(A)** Heat maps of the average expression levels (left panel) and their relative divergences (right panel) of the cancer-related genes. The color key is shown in the bottom margin. **(B)** Expression levels of representative genes in the indicated cell lines. Statistical significance for the differences was evaluated by *t*-test for the average expression levels (red) or by f-test for relative divergences (blue).

We further examined the expression patterns of the 25 representative cancer-driver or tumor-suppressor genes, which were selected from recent papers on clinical genome sequencing of lung cancers (we call them 'cancer-related genes' hereafter; the expression patterns of five representative cancer-related genes are shown in Table 2) [21-23]. Additionally, for these genes, the relative divergences converged to 1.0 for the genes with expression levels >5 rpkm (Additional file 6). Again, we observed that each cell had unique expression patterns not only for average expression levels but also for relative divergences for functionally important genes (Figure 4A).

**Table 2:** **Gene expression variations for representative cancer-related genes in single cells of different cell lines**

	**LC2/ad**	**LC2/ad (rep)**	**LC2/ad-R**	**PC-9**	**VMRC-LCD**
EGFR	13 ± 14	17 ± 15	12 ± 14	56 ± 34	0.03 ± 0.1
RET	1.9 ± 5	2.0 ± 4	1.6 ± 5	0.005 ± 0.015	1.0 ± 7
MYC	99 ± 117	84 ± 95	179 ± 123	23 ± 23	0.05 ± 0.2
KRAS	29 ± 17	27 ± 19	17 ± 14	21 ± 15	26 ± 19
TP53	22 ± 15	25 ± 14	105 ± 94	126 ± 43	14 ± 9

Expression level is presented as average ± standard deviation.

To further investigate the biological meaning of the observed unique patterns of relative divergences based on genes and cell lines, we considered whether each line carries any known driver mutations in the corresponding genes. Whole-genome sequencing of these cell lines showed that neither LC2/ad, PC-9 nor VMRC-LCD is known to carry any driver mutations in the KRAS gene (Figure S10D in Additional file 1). The expression levels of the KRAS gene and their variation were almost similar between the cell lines (Figure 4B). However, for the EGFR gene, the average gene expression levels were 4.4 times and >1,000 times higher in PC-9, which carries a driver mutation, than in LC2/ad and VMRC-LCD, respectively, both of which do not possess such a driver mutation. In PC-9, the relative divergence of the EGFR gene was 1.8 times and 5 times narrower than in LC2/ad and VMRC-LCD, respectively (see Figure S4C in Additional file 1 for the validation analysis). In the case of the RET gene, however, for which LC2/ad carries a gene fusion, the highest expression level and the narrowest divergence were observed in LC2/ad. It is tempting to speculate that the EGFR gene in PC-9 and the RET gene in LC2/ad may be under stricter selection pressures. Thus, the increased expression levels and the narrow divergence for the corresponding genes would be natural consequences in these cell lines.

The MYC gene provides another unique example. The average expression level of this gene was highest in LC2/ad, 4.4 times higher than in PC-9; however, the relative divergence was almost equivalent between them (less than 1.2-fold difference). In LC2/ad, the MYC gene was found to be genomically amplified (Figure S12 in Additional file 1). Similarly, in the case of the CCNC gene, for which genome amplification was observed solely in VMRC-LCD, expression levels were 6.0 and 4.9 times higher but with similar levels of relative divergence (1.4- and 1.9-fold difference) compared with PC-9 and LC2/ad, respectively. Relative divergences may reflect distinct mechanisms of up-regulation of gene expression. In either case, it should be particularly important to further investigate how the observed divergence in gene expression is realized through transcriptional mechanisms and to what extent these mechanisms contribute to characteristic phenotypic differences in each cell line.

### Changes in gene expression patterns in response to vandetanib stimulation

To examine how gene expression divergences vary in response to a molecular target drug, we conducted a similar single-cell RNA-Seq analysis using LC2/ad treated with vandetanib (1 μM for 6 hours; IC50 = 0.32 μM; Figure S13 in Additional file 1). We also utilized an LC2/ad-derived cell line, LC2/ad-R, which has acquired resistance to vandetanib (IC50 = 1.13 μM; Figure S13 in Additional file 1), in a similar analysis (Table 1; statistics and validation analyses of the RNA-Seq are shown in Figure S11D in Additional file 1 and in Additional file 2). Whole-genome sequencing of LC2/ad-R showed that essentially no driver mutations in cancer-related genes, such as those in the EGFR and KRAS genes, were newly acquired in LC2/ad-R (Figure S10E in Additional file 1).

**Figure 5:**
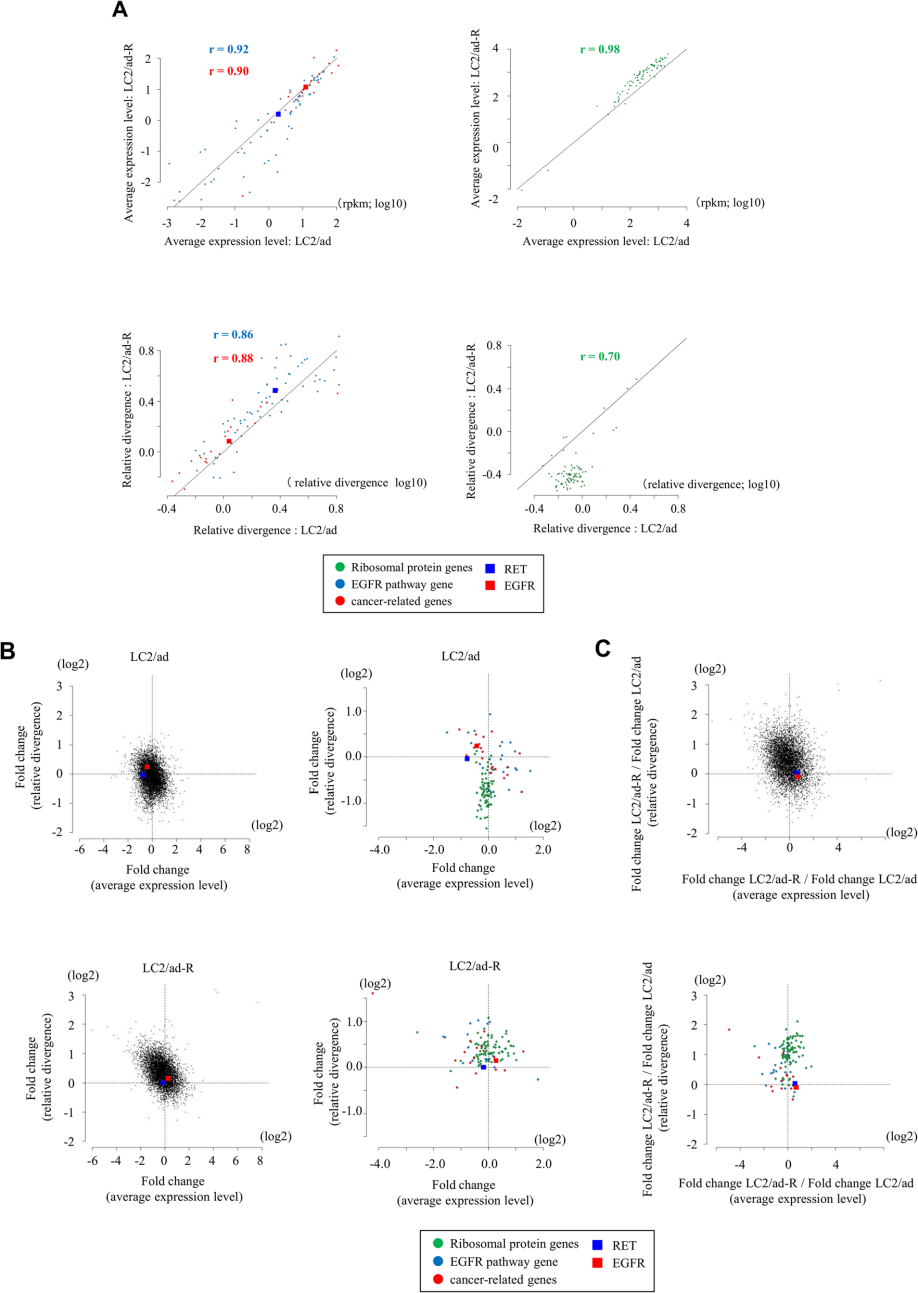
Expression changes in response to anti-cancer drug stimulation. **(A)** Correlation of the average expression levels and the relative divergences between LC2/ad and LC2/ad-R cells for EGFR pathway genes (blue), caner-related genes (red) and ribosomal protein genes (green). Pearson’s correlation coefficients are shown in the plots. The plots of the EGFR and RET genes are highlighted by red and blue boxes, respectively. **(B)** Gene expression changes in response to vandetanib treatment in LC2/ad (upper panel) and LC2/ad-R (lower panel) cells. Each gene, plots show fold changes in the average expression levels (x-axis) and the relative divergence. The dotted lines represent the values that were unchanged (fold = 1). Right panels show plots for ribosomal protein genes, the EGFR pathway genes and the cancer-related genes. The color key is as in **(A)**. **(C)** Relative fold changes in average expression levels (x-axis) and relative divergences (y-axis) for LC2/ad and LC2/ad-R cells. The dotted lines indicate the values that were unchanged between these cell lines. The lower panel shows ribosomal protein genes, the EGFR pathway genes and the cancer-related genes. The color key is as in **(A)**.

First, we compared the expression patterns between LC2/ad and LC2/ad-R without stimulation. We found that the average expression levels of many of the so-called house-keeping genes [19,24], represented by ribosomal protein genes (right panels in Figure 5A; see Figure S14 in Additional file 1 for further details; see Figure S15 in Additional file 1 and Additional file 7 for changes in relative divergence in other house-keeping genes), were similar between them. However, their relative divergences were significantly lower in the LC2/ad-R cells. Because LC2/ad-R cells were derived from a subpopulation of parental LC2/ad cells, their expression patterns may be originally more homogeneous than parental cells. Unlike the case of ribosomal protein genes, both the expression levels and the relative divergences of the EGFR pathway genes and cancer-related genes were similar between these cell lines (left panels in Figure 5A).

Next, we compared the fold changes of the average expression levels in response to vandetanib treatment. We selected the genes for which average expression levels changed more than two-fold in response to the vandetanib treatment. We identified 1,202 such genes (457 genes that were induced and 745 genes that were repressed) in LC2/ad and 2,037 such genes (539 genes that were induced and 1,498 genes that were repressed) in LC2/ad-R (Figure S16 in Additional file 1). A wide variety of genes were included, likely reflecting the fact that the anti-cancer drug treatment affected various signaling pathways in both LC2/ad and LC2/ad-R (Table S10 in Additional file 1). Additionally, distinct patterns of alternations in gene expression were observed between LC2/ad and LC2/ad-R (Figure S16 in Additional file 1), likely reflecting diverse responses in these cell lines to the vandetanib treatment.

We found that, compared with the parental cells, average expression levels generally changed more in LC2/ad-R (Figure 5B; Figure S16 in Additional file 1). LC2/ad-R cells may have acquired the ability to plastically change their transcriptome regulation in response to the vandetanib treatment. We also examined fold changes of the relative divergences. We found that changes in the relative divergences were more significant in the parental LC2/ad line. In this case, for many of the house-keeping genes, as exemplified by the ribosomal protein genes (upper panel in Figure 5B), the relative divergences were greatly reduced in LC2/ad, as if the cells lose diversity in response to the drug treatment. However, such reductions in relative divergences were not observed in LC2/ad-R. Rather, changes were sometimes slightly induced even when the average expression levels were unchanged (lower panel in Figure 5B). In summary, LC2/ad-R showed reduced average gene expression and LC2/ad showed reduced relative divergence (upper panel in Figure 5C; note that the dots are enriched in the upper left part of the plots).

In particular, for EGFR pathway genes and cancer-related genes, typical alterations in average expression levels or relative divergences in response to vandetanib were not significant (right panels in Figure 5B; lower panel in Figure 5C). Expression levels and relative divergences of the EGFR and RET genes, which are direct targets of vandetanib [25], remained unchanged both in LC2/ad and LC2/ad-R. Cellular survival of parental LC2/ad should be heavily dependent on these genes. Therefore, their expression may have been robustly regulated, even with some allowance for diversity among different cells, but could not be altered by the drug treatment. Although LC2/ad-R is not dependent on these genes, and vandetanib inhibited their activities as tyrosine kinases, such changes were not reflected as a change in the transcriptional program due to their rigid transcriptional regulation, which may be due to the inherent nature of this cell type. For genes directly related to cancers, such as cancer drivers, distinct types of selective pressure may have been exerted by other genes.

### Gene expression patterns of single cells

**Figure 6:**
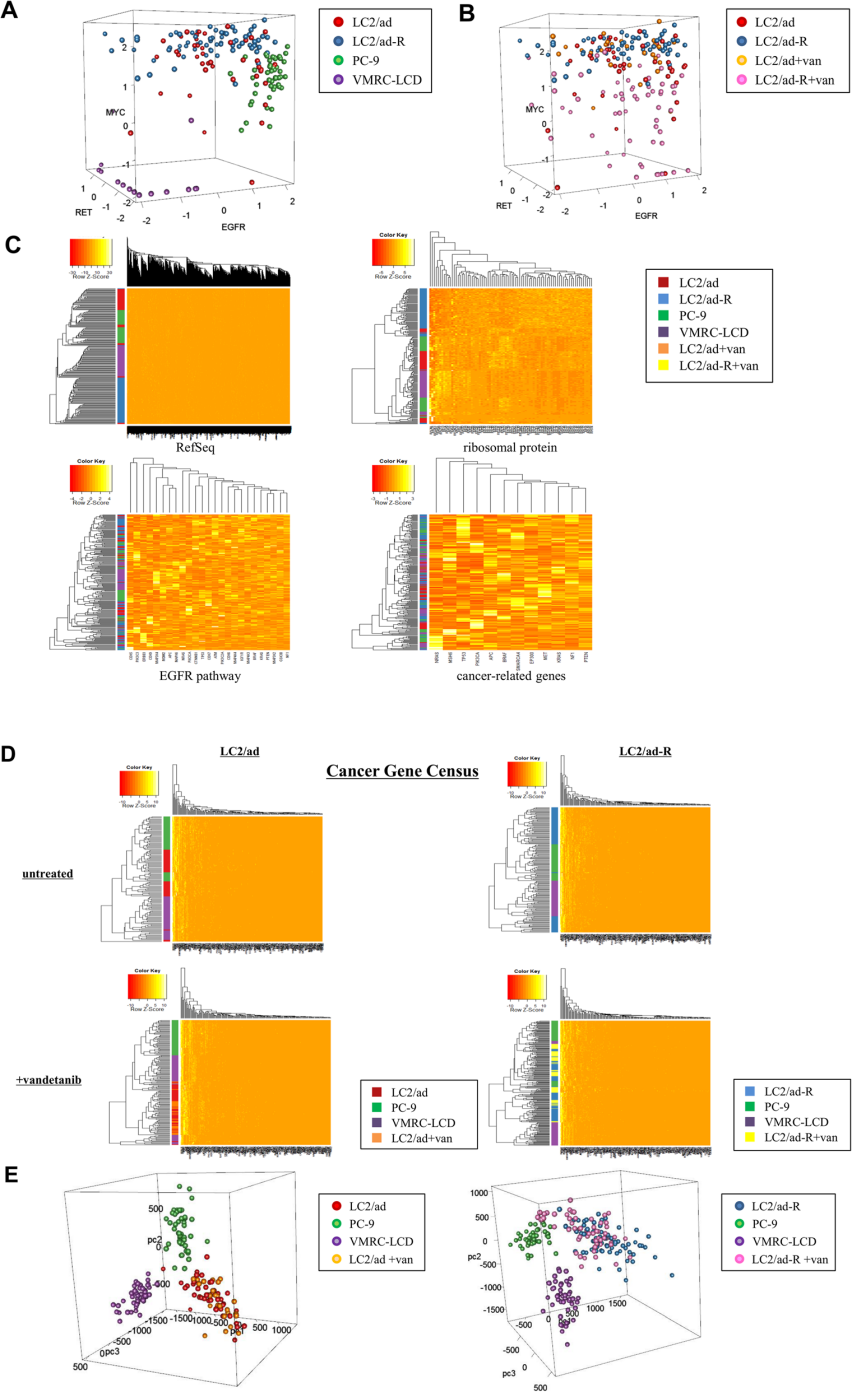
Gene expression patterns in a given cell. **(A)** Individual single cells of different cell lines are plotted according to expression levels of the EGFR, MYC and RET genes. The color key for the cell lines is shown to the right. **(B)** Results of a similar analysis as in (A) but for LC2/ad and LC2/ad-R cells treated with or vandetanib or not. Notable expression changes observed for LC2/ad-R cells are indicated by pink circles. **(C)** Hierarchal clustering analyses were conducted using the indicated groups of genes. Clusters of individual cells are represented in the left margin of the heat maps. The color key for the cells is shown to the right. **(D)** Results of a similar analysis as (C) but for Cancer Gene Census genes. The color key for the cell lines is shown to the right of the heat maps. **(E)** Results of the principle component analysis for Cancer Gene Census genes in LC2/ad (left panel) and LC2/ad-R (right panel) cells. Color keys for the cell lines are shown to the right.

In addition to investigating gene expression diversity between individual cells, we wished to analyze the expression levels of genes and their mutual relations within an individual cell. We plotted individual cells of each cell type according to the expression levels of the EGFR, MYC and RET genes, using 205 cells (43, 70, 46 and 46 cells from LC2/ad, LC2/ad-R, PC-9 and VMRC-LCD, respectively). As expected, individual cells formed clusters depending on parent cell types (Figure 6A). However, we also observed that a number of cells deviated from the center of each cluster, suggesting heterogeneity within the populations. We also conducted similar analysis using vandetanib-treated cells, with a total of 199 cells (43, 70, 28 and 58 cells from LC2/ad, LC2/ad-R, LC2/ad + vandetanib and LC2/ad-R + vandetanib, respectively). We observed that heterogeneity was more prominent for LC2/ad-R cells than for the parental LC2/ad cells (Figure 6B). Particularly, those differences were most significant for the expression level of MYC, suggesting that MYC may play a pivotal role in differentiating these cell lines.

We also conducted a global clustering analysis of gene expression using 205 cells. As expected, when we used all genes for the clustering, we found that individual cells formed clusters depending on their parent cell types (vertical color bar in Figure 6C). Similar results were obtained when we used the 88 ribosomal protein genes to perform the clustering. By contrast, when we examined the cancer-related genes or the EGFR pathway genes, the individual cells of different cell types did not form significant clusters, suggesting that the expression patterns of these groups of genes were intrinsically heterogeneous even in the untreated state. Interestingly, when we used the 'Cancer Gene Census' genes [26], which is a catalogue of genes associated with carcinogenesis in various cancer types, the clusters were still clear between the different cell lines. However, when we considered the data for LC2/ad and LC2/ad-R cells treated with vandetanib, we found that the clusters were occasionally disordered across their originating cell types (Figure 6D; see Figure S17 in Additional file 1 for gene expression changes based on drug treatment). Interestingly, while the LC2/ad + vandetanib cells overlapped with LC2/ad cells, LC2/ad-R + vandetanib cells tended to form distinct clusters from LC2/ad-R cells, as if the parental cells responded in a random manner, while the response of the derivative cells was deterministic to some extent (see Figure S18 in Additional file 1 for distributions of cluster sizes and statistical significance of the difference between LC2/ad and LC2/ad-R).

Furthermore, in the case of the Cancer Gene Census genes, we conducted a principle component analysis (Figure 6E). We found that the LC2/ad-R cells, rather than parental LC2/ad cells, formed relatively tight clusters in both the untreated and the vandetanib-treated states. Interestingly, some of the cell clusters of LC2/ad-R + vandetanib came closer to or partially overlapped clusters of PC-9. These genes may have evolved expression patterns resembling those of PC-9 to avoid the effect of vandetanib on LC2/ad cells. Various patterns of gene expression diversity, some of which are apparent and some of which are latent, may collectively provide a versatile base from which drug resistance could emerge.

## Conclusions

In this study, we show gene expression diversity in lung adenocarcinoma cell lines by employing single-cell RNA-Seq analysis. To our knowledge, this is the first study that has described the heterogeneity of cancer cells at the transcriptome level.

We believe that it is unlikely that the results obtained in this study derive from typical technical errors. However, we could not exclude all of the possibilities of false observations. First, we did not use cells that were synchronized for the cell cycle. Perhaps consistently, cell cycle-related genes were enriched in the pathways with diverse expression patterns (Figure 2H). Neither could we completely control the micro-environment for each cell. Therefore, the observed differences in the gene expression patterns may reflect the differential representation of the population of cells with regard to these factors. Second, we could not evaluate the influence of the systematic bias that was imposed during library construction. Inevitably, heavy amplification of the templates was performed for this analysis (also see Figure 1D for a comparison between the average of the single cells and the bulk cells). Noise could also be induced at the initial step, including reverse-transcription, which is particularly difficult to evaluate in the present system. Once harvested, the same cell cannot be used for any validation analyses. In this study, we evaluated variation in the spike-in controls, which were added as RNA at the cell lysis step (Figure 1A). We excluded cells where the tag counts of any of three spike-in controls deviated by more than two standard deviations from the average values from the following analyses. Also, we examined and found the observed average expression levels and relative divergences were reasonably reproducible between two independent experiments (r = 0.93 and 0.82, respectively; Figures 1D (second panel) and 2D). These results should account for overall technical errors, including those induced during the reverse transcription stage. Nevertheless, it remains unclear to what extent they truly represent the collective information within a cell *in vivo*, even though they were reproducible (Figure S2B in Additional file 1). Lastly, but no less importantly, we could not attribute the cause of the observed divergences to newly acquired novel genomic mutations or other epigenomic alterations. For each of these issues, further extensive analyses are required.

In spite of several drawbacks, we believe that this study should lay the foundations for single-cell analysis of cancer cells. Indeed, we have found that gene expression was highly diverse between individual cells, which is characteristic of the genes, pathways and cell lines. We also observed that there is a general tendency that lowly expressed genes show high divergence. This tendency was not systematic, however, since we observed that the degree of divergence varied between genes even when their average expression levels were almost the same. We were not able to identify molecular mechanisms underlying these characteristic divergences, though it is tempting to speculate that there are distinctive determinants depending on the involved signaling pathways or gene expression regulatory mechanisms. It is also possible that high relative divergences in lowly expressed genes derived from the inevitably stochastic nature of the transcriptional machineries. Additionally, we examined and found that not only average gene expression levels but also their relative divergences were changed in response to drug treatment. Furthermore, we unexpectedly found that the expression patterns of several cancer-related genes within a single cell are occasionally more diverse beyond the borders of its originating cell type. In particular, Cancer Gene Census genes showed a unique pattern; such divergence initially became apparent when the cells were treated with a molecular target drug.

Various types of potential divergence in transcriptome regulation may collectively serve as a reservoir for cells to eventually acquire drug resistance. To further clarify this possibility, it is necessary to investigate not only average gene expression levels and their fold changes in response to anti-cancer drug treatment but also the variance of these genes between individual cells. We believe that further extensive single-cell transcriptome analysis using more cell types in various environmental conditions will bring invaluable insight for understanding how diverse phenotypes of cancer cells emerge from a given population of cancer cells.

## Materials and methods

### Data availability

All of the sequence data used in the present study have been registered in the DNA Data Bank of Japan under accession numbers DRA001287 and DRA002730. The graphical view for each gene is also available from our web site at [27].

### Cell culture and sequencing

The LC2/ad and PC-9 cell lines were acquired from the RIKEN Bio Resource Center (catalogue numbers RCB0440 and RCB4455, respectively). The VMRC-LCD cell line was provided by the Japanese Collection of Research Bioresources (catalogue number JCRB0814). The LC2/ad-R cell line was provided on request. Cell culture mediums were prepared using Dulbecco’s modified Eagle’s medium (DMEM 2, Nissui Pharmaceutical, Tokyo, Japan) for LC2/ad and LC2/ad-R, RPMI medium (RPMI 1640 2, Nissui Pharmaceutical) for PC-9 or Eagle’s minimal essential medium (EMEM 1, Nissui Pharmaceutical) for VMRC-LCD, supplemented with 10% fetal bovine serum, MEM Non-essential Amino acid solution (catalogue number M7145, Sigma-Aldrich, St Louis, MO, USA) and Antibiotic-Antimycotic (catalogue number 15240–062, Gibco/Life Technologies, Carlsbad, CA, USA). The LC2/ad and LC2/ad-R cell lines were cultured in collagen type I-coated dishes (IWAKI, AGC Techno Glass, Tokyo, Japan). For vandetanib (catalogue number S1046, Selleck Chemicals, Houston, TX, USA) treatment, vandetanib was administered to the culture medium at a final concentration of 1 μM. Six hours after the drug treatment, cells were harvested. For each experiment, 10^6^ cells were harvested and used for the single-cell RNA-Seq analyses using the C1 system (Fluidigm, South San Francisco, CA, USA). RNA-Seq libraries were constructed according to manufacturers' instructions as follows. Briefly, 96 cells were captured in the flow cells and separated into independent chambers. First-strand cDNA was synthesized and further amplified using the SMARTer system (Clontech, Mountain View, CA, USA). Illumina sequencing libraries were constructed using Nextera XT DNA Sample Preparation kit (Illumina, San Diego, CA, USA). After evaluation of the quality and quantity of the constructed RNA-Seq libraries using a BioAnalyzer (Agilent Technologies, Santa Clara, CA, USA), sequencing was performed on the HiSeq2500 platform with a 97-base paired-end read. Generated RNA-Seq tags were mapped to the reference human genome (hg19; UCSC) using ELAND. Sequences that mapped to the unique genomic positions allowing two base mismatches were used. RNA-Seq tags that spanned the known splice junctions were also considered. The number of RNA-Seq libraries and RNA-Seq tags used for the analyses are shown in Table 1. The primers for quantitative RT-PCR validation analyses of 85 genes (a standard validation dataset from Fluidigm) were provided as the Human Gx performance panel (P/N 100-5396) and the raw data for individual genes are shown in Additional file 3. These 85 genes were selected from the genes having diverse expression levels and are likely to be expressed in a wide range of cell types [24,28].

### Computational procedures

RNA-Seq tag counts were calculated as parts per million mapped tags per kilobase RNA (rpkm). The average expression level of a given gene was calculated as an average of the population of cells. Relative divergence was calculated as standard deviation divided by average gene expression level. The statistical significance of the differences was evaluated by the indicated methods. To select genes showing diverse gene expression, the genes were ordered according to their relative divergences. To select KEGG pathways for which the genes with diverse expression were enriched, a gene with a similar expression level (a less than two-fold difference) was randomly selected for each of the genes. One thousand genes were randomly selected when the genes satisfying this criterion exceeded 1,000 genes. Relative divergences between individual cells were compared between the examined gene and the control genes. Statistical deviation of the relative divergence of the examined gene against the background distribution of the control genes was evaluated by f-test and genes giving *P* < 0.05 were selected. Enrichment of the selected more or less diverse genes in a particular KEGG pathway was evaluated by Fisher’s test. The cases were selected when the calculated *P* < 0.05. To calculate the fold changes in average gene expression levels and their relative standard deviations, genes showing average expression levels of >5 rpkm were used, unless noted otherwise. For clustering, a hierarchal clustering program in the bioconductor package of R [29] was used. Cancer-related genes were selected manually based on [21-23]. The list of Cancer Gene Census genes were obtained from the Cancer Gene Census [26].

To investigate the genomic status of the cancer cell lines, whole-genome sequences (registered in the DNA Data Bank of Japan under accession number DRA001859) [20] were mapped to a human reference genome (hg19, UCSC) using BWA [30] and SAMtools [31] and visualized by IGV [32,33]. To compare mutations in the LC2/ad and LC2/ad-R cell lines, single nucleotide variants (SNVs) and insertion/deletions (indels) were detected using GATK [34,35] and annotated using Polyphen-2 [36,37] and in-house Perl scripts. To remove germline variants and select somatic mutations, we used information provided from the 1000 Genomes Project, the NHLBI Exome Sequencing Project, NCBI dbSNP build 137, COSMIC (v59) and in-house Japanese normal tissues [38-42].

## Additional files

Additional file 1: Figure S1. Preparation of single-cell RNA-Seq libraries. **Figure S2.** Validation analyses on sequence depth and re-amplification of the templates. **Figure S3.** RNA-Seq tags representing known driver mutations. **Figure S4.** Validation analysis using real time RT-PCR assays in individual cells of PC-9. **Figure S5** Dependency of the relative divergences on the sequence depth for the spike-in controls. **Figure S6.** Dependency of the relative divergences on the sequence depth for the gene of varying average expression levels. **Figure S7.** Dependency of the relative divergences on the sequence depth for the cancer-related genes. **Figure S8.** Relations between the sequence depths and the number of tags in respective genes. **Figure S9.** Dependency of the calculated relative divergence on the varying numbers of cells. **Figure S10.** Information on whole-genome sequences of the cell lines. **Figure S11.** RNA-Seq tags generated from different cell lines. **Figure S12.** Amplifications detected by whole-genome sequences. **Figure S13.** Drug response of LC2/ad and LC2/ad-R cells. **Figure S14.** Comparison of the gene expression differences between LC2/ad and LC2/ad-R. **Figure S15.** Relative divergences of other house-keeping genes in LC2/ad and LC2/ad-R. **Figure S16.** Gene expression changes in response to vandetanib. **Figure S17.** Gene expression changes of Cancer Gene Census genes. **Figure S18.** Size of the clusters in LC2/ad and LC2/ad-R stimulated with vandetanib. **Table S2.** Comparison of RNA-Seq statistics between bulk and single-cell libraries. **Table S4.** Primer sequences for real time RT-PCR assays of 13 genes. **Table S6.** Gene Ontology terms and KEGG pathways enriched for genes with expression divergences that were notable in PC-9 and VMRC-LCD. **Table S10.** Gene Ontology terms and KEGG pathways enriched for genes that showed fold inductions of ≥2 or ≤0.5 with regard to average gene expression levels and relative divergences in response to vandetanib. Additional file 2: Table S1. Detailed statistics of the RNA-Seq tags generated and used in the present study. Additional file 3: Table S3. A list of 85 genes using quantitative RT-PCR. Additional file 4: Table S5. Genes with significantly diverse **(A)** or less diverse **(B)** expression patterns. Additional file 5: Table S7. Average expression levels and relative variations of the genes belonging to the EGFR pathway. Additional file 6: Table S8. Average gene expression levels and relative divergences for the cancer-related genes appearing in Figure 4A. Additional file 7: Table S9. Lists of genes used for the analysis in **Figure S14.** in Additional file 1. **(A**-**E)** Genes in the indicated KEGG pathways were selected from the 560 house-keeping genes previously reported [24]. **(F)** The selected genes used for real time RT-PCR assays are also shown.

## Abbreviations

KEGG: Kyoto Encyclopedia of Genes and Genomes
rpkm: reads per million tags per kilobase mRNA
