# Supplementary material for: Single-cell analysis of lung adenocarcinoma cell lines reveals diverse expression patterns of individual cells invoked by a molecular target drug treatment
Source: Genome Biol. 2015 Apr 3;16(1):66. doi: 10.1186/s13059-015-0636-y (PMC4450998; doi:10.1186/s13059-015-0636-y)
Supplement: Additional file 1: Figure S1. — Preparation of single-cell RNA-Seq libraries. Figure S2. Validation analyses on sequence depth and re-amplification of the templates. Figure S3. RNA-Seq tags representing known driver mutations. Figure S4. Validation analysis using real time RT-PCR assays in individual cells of PC-9. Figure S5 Dependency of the relative divergences on the sequence depth for the spike-in controls. Figure S6. Dependency of the relative divergences on the sequence depth for the gene of varying average expression levels. Figure S7. Dependency of the relative divergences on the sequence depth for the cancer-related genes. Figure S8. Relations between the sequence depths and the number of tags in respective genes. Figure S9. Dependency of the calculated relative divergence on the varying numbers of cells. Figure S10. Information on whole-genome sequences of the cell lines. Figure S11. RNA-Seq tags generated from different cell lines. Figure S12. Amplifications detected by whole-genome sequences. Figure S13. Drug response of LC2/ad and LC2/ad-R cells. Figure S14. Comparison of the gene expression differences between LC2/ad and LC2/ad-R. Figure S15. Relative divergences of other house-keeping genes in LC2/ad and LC2/ad-R. Figure S16. Gene expression changes in response to vandetanib. Figure S17. Gene expression changes of Cancer Gene Census genes. Figure S18. Size of the clusters in LC2/ad and LC2/ad-R stimulated with vandetanib. Table S2. Comparison of RNA-Seq statistics between bulk and single-cell libraries. Table S4. Primer sequences for real time RT-PCR assays of 13 genes. Table S6. Gene Ontology terms and KEGG pathways enriched for genes with expression divergences that were notable in PC-9 and VMRC-LCD. Table S10. Gene Ontology terms and KEGG pathways enriched for genes that showed fold inductions of ≥2 or ≤0.5 with regard to average gene expression levels and relative divergences in response to vandetanib. [file 13059_2015_636_MOESM1_ESM.pdf]

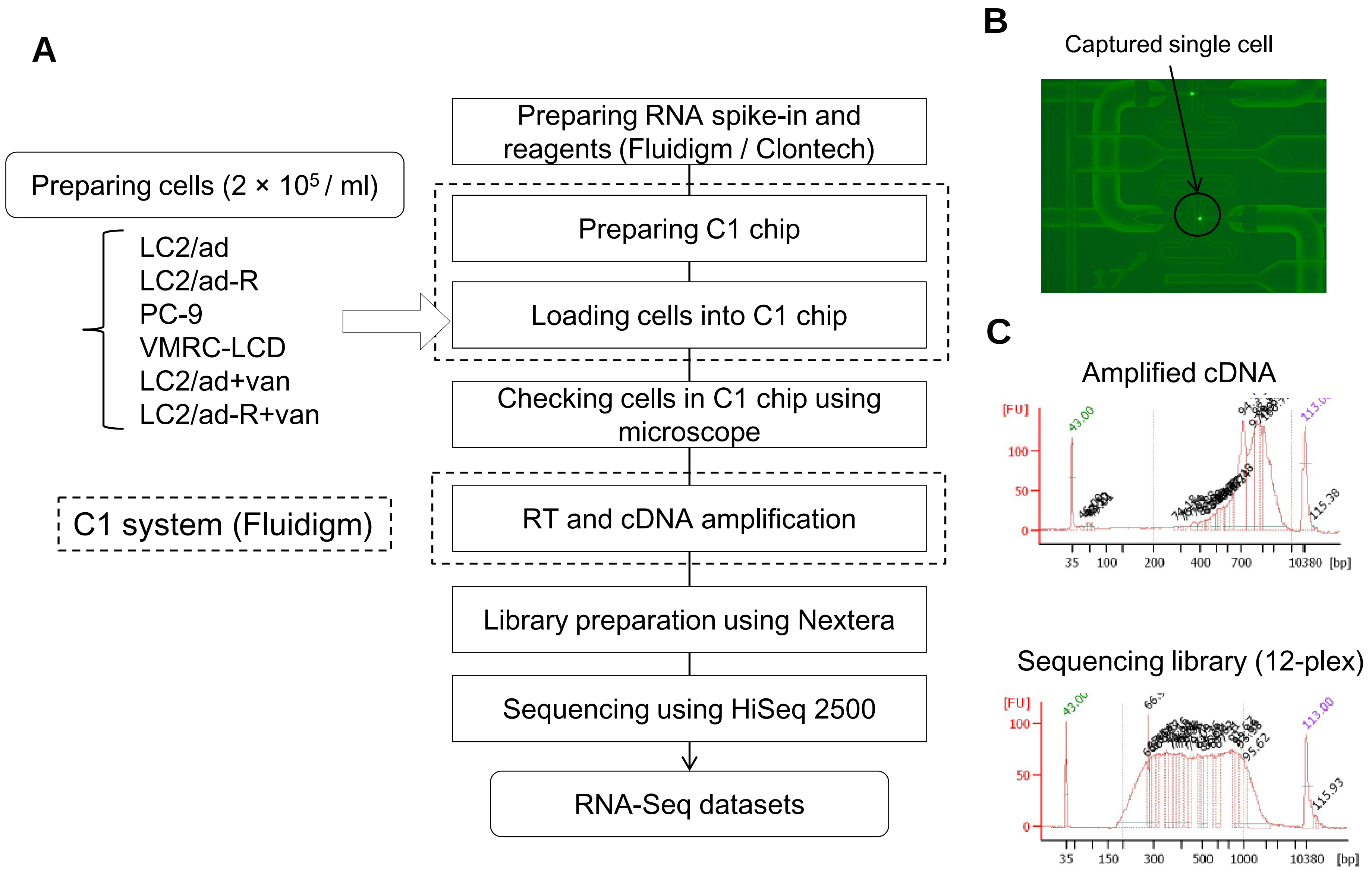

A
Preparing cells (2 × 10⁵ / ml)
LC2/ad
LC2/ad-R
PC-9
VMRC-LCD
LC2/ad+van
LC2/ad-R+van
C1 system (Fluidigm)
Preparing RNA spike-in and reagents (Fluidigm / Clontech)
Preparing C1 chip
Loading cells into C1 chip
Checking cells in C1 chip using microscope
RT and cDNA amplification
Library preparation using Nextera
Sequencing using HiSeq 2500
RNA-Seq datasets
B
Captured single cell
C
Amplified cDNA
[FU]
100
50
0
35
100
200
400
700
10380
[bp]
Sequencing library (12-plex)
[FU]
100
50
0
35
150
300
500
1000
10380
[bp]

Figure S1

Additional file 1

Figure S1 Preparation of single-cell RNA-Seq libraries. (**A**) Work flow and quality control of the intermediate products for the RNA-Seq library construction. (**B**) Capture image of an individual cell. (**C**) For a representative case, BioAnalyzer images of the cDNAs and the final template are shown in the upper and lower panel, respectively**.**

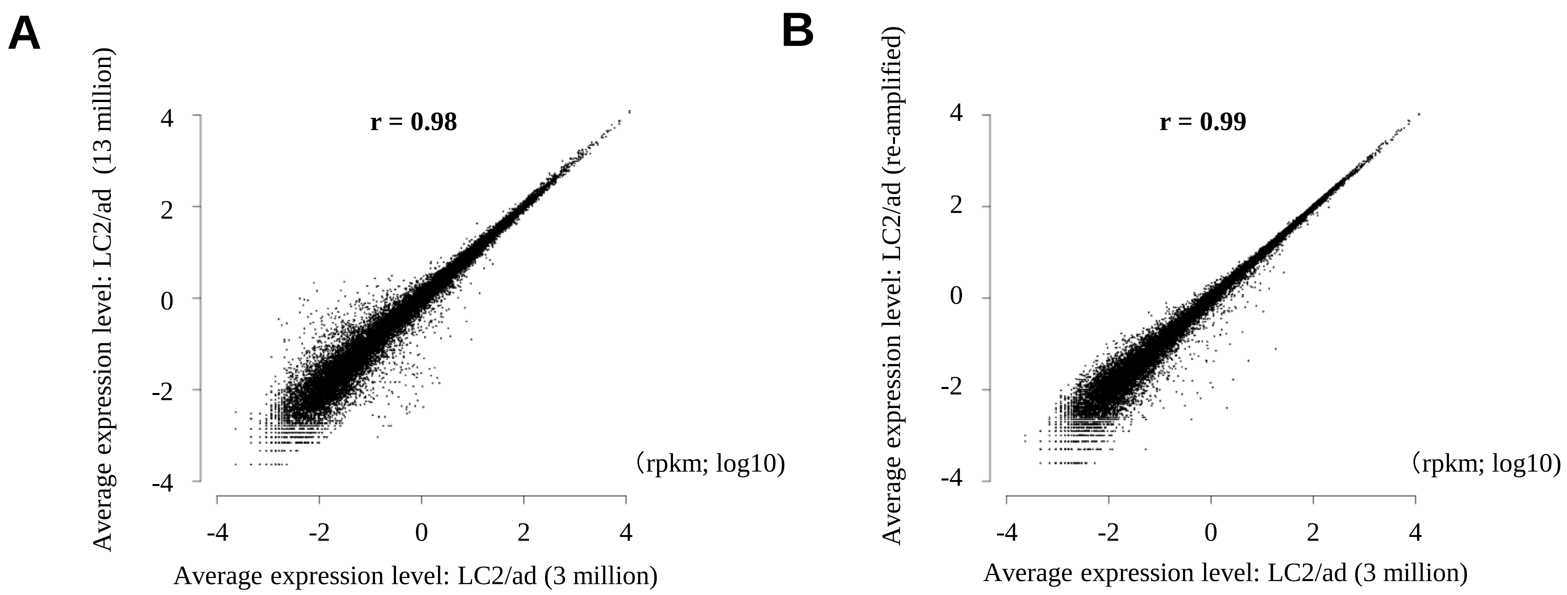
A
B
r = 0.98
r = 0.99
Average expression level: LC2/ad (13 million)
Average expression level: LC2/ad (re-amplified)
Average expression level: LC2/ad (3 million)
Average expression level: LC2/ad (3 million)
(rpkm; log10)
(rpkm; log10)
4
2
0
-2
-4

Figure S2

Additional file 1

Figure S2 Validation analyses on the sequence depth and re-amplification of the templates. (**A**) Relations of the average gene expression levels calculated based on the original dataset (3 million tags per single cell on average) and increased sequence depth (13 million tags per single cell on average). (**B**) Relations of the average gene expression levels calculated based on the original dataset and the dataset obtained from re-amplified RNA-Seq library at the similar sequence depth. Pearson's correlation coefficients for the respective panels are shown in the plot areas.

A

	LC2/ad at 3million		LC2/ad at 13million		PC-9	
	total tag	#expressing cells	total tag	#expressing cells	total tag	#expressing cells
CCDC6-RET	18	2	89	7	0	0
EGFR del	0	0	0	0	514	14

B

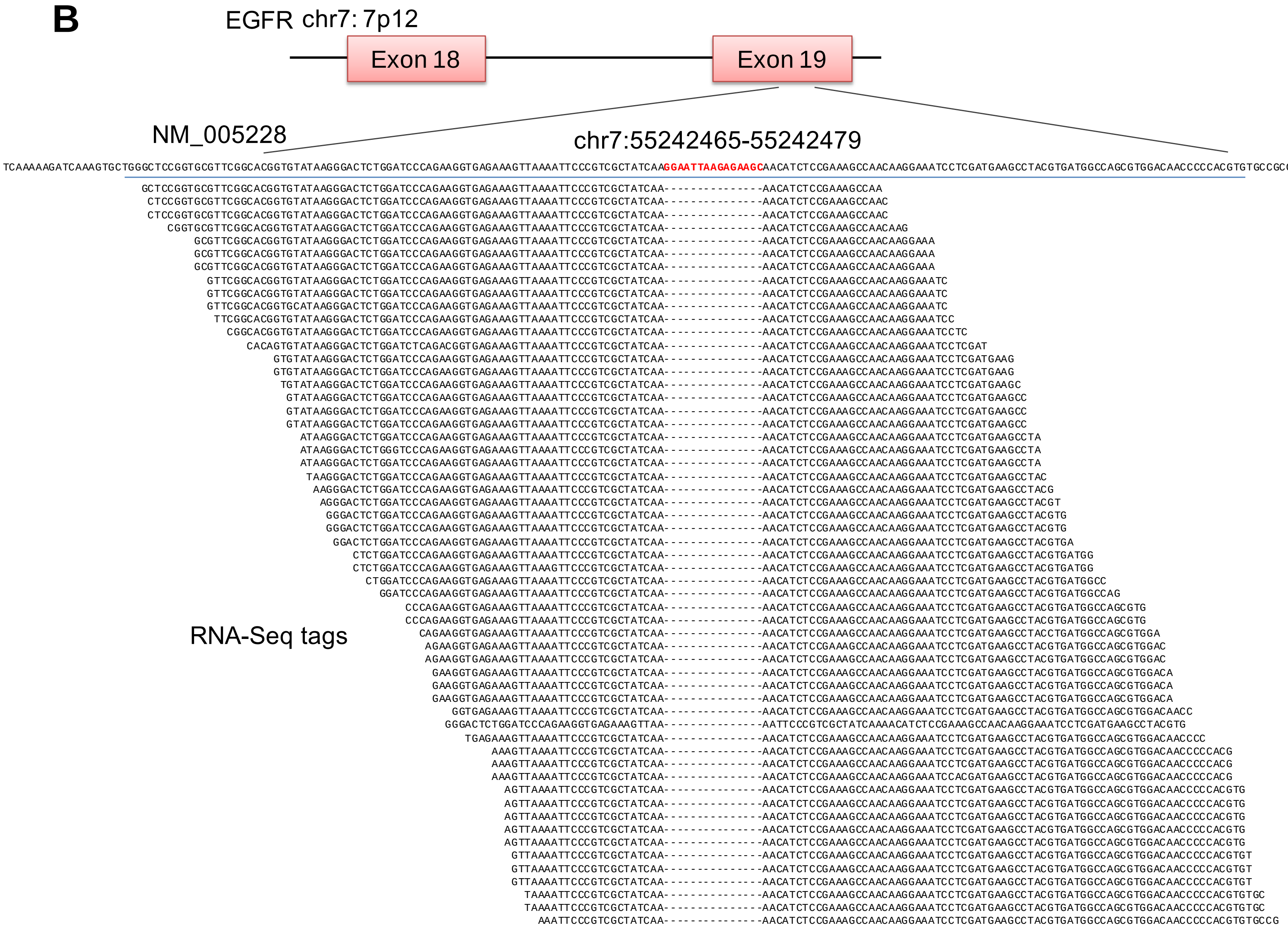

Figure S3

Additional file 1

Figure S3 RNA-Seq tags representing known driver mutations. (**A**) Number of tags and the number of the cells in which the corresponding tags were identified are shown. For LC2/ad, these numbers increased with increasing sequence depth from 3 million tags to 13 million tags for a single cell on average. (**B**) Example of the sequence tags which spanned known deletion sites of the EGFR genes (E746_A750del; chr7: 55242465–55242479, −GGAATTAAGAGAAGC) .

Reference:

Sharma SV, Bell DW, Settleman J, Haber DA. Epidermal growth factor receptor mutations in lung cancer. Nat Rev Cancer. 2007;7:169–81.

A

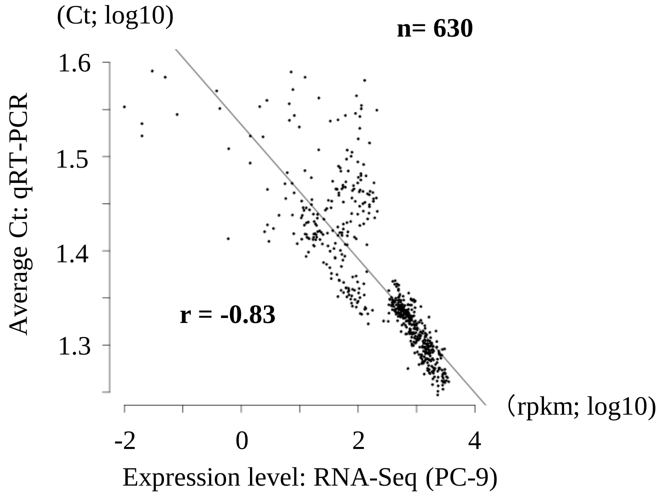

B

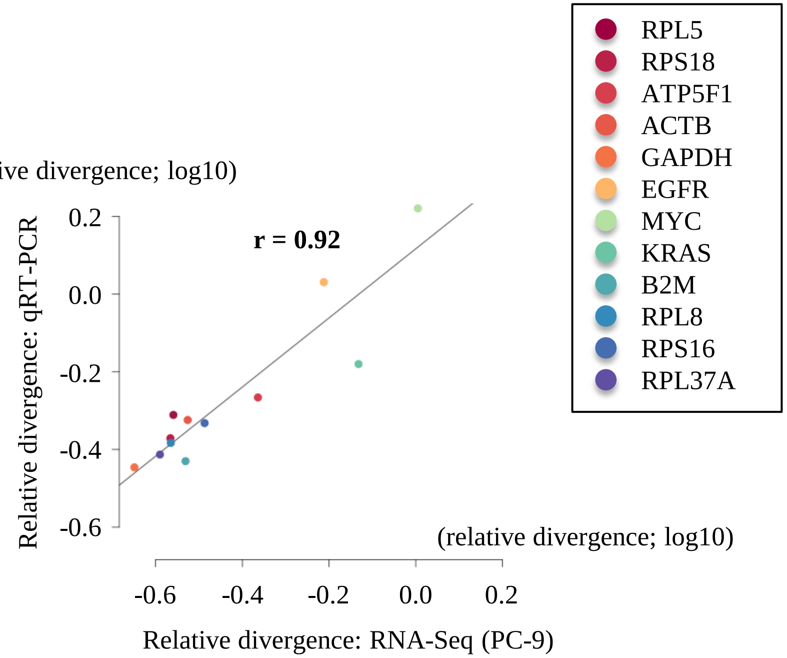

C

	RNA-Seq	Real time RT-PCR
LC2/ad	1.8	2.1
PC-9	1.0	1.0

(Fold change of relative divergence in EGFR)

Figure S4

Additional file 1

Figure S4 Validation analysis using real time RT-PCR assays in individual cells of PC-9. The examined genes and PCR primers used for the analyses are same in the case of LC2/ad (Figure 2E and F). Correlation between real time RT-PCR assays and single-cell RNA-Seq are shown for the expression levels of individual cells (**A**) and relative divergences (**B**). (**C**) Fold change of the relative divergence for EGFR, which were calculated based on the results of the RNA-Seq (first column) and real time RT-PCR (second column) methods. Fold changes were calculated with the values of PC-9 being designated as 1.

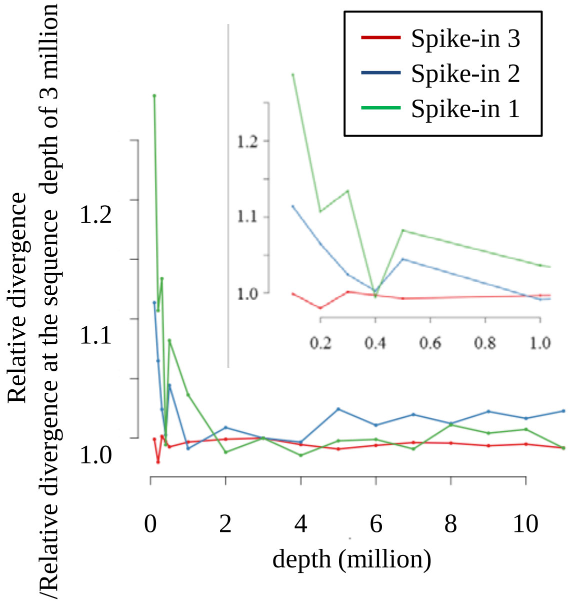

Spike-in 3
Spike-in 2
Spike-in 1
Relative divergence
/Relative divergence at the sequence depth of 3 million
1.2
1.1
1.0
0
2
4
6
8
10
depth (million)
1.2
1.1
1.0
0.2
0.4
0.6
0.8
1.0

Figure S5

Additional file 1

Figure S5 Dependency of the relative divergences on the sequence depth for the spike-in controls. Dependency of the calculated relative divergence on the varying sequence depth per cell (x-axis) is shown for each of the spike-in controls. Relative deviations of the calculated relative divergences compared with those at the sequence depth of three million per cell were calculated at the indicated sequence depth. Inset represents the magnification of the main plot at the region of small values of x-axis.

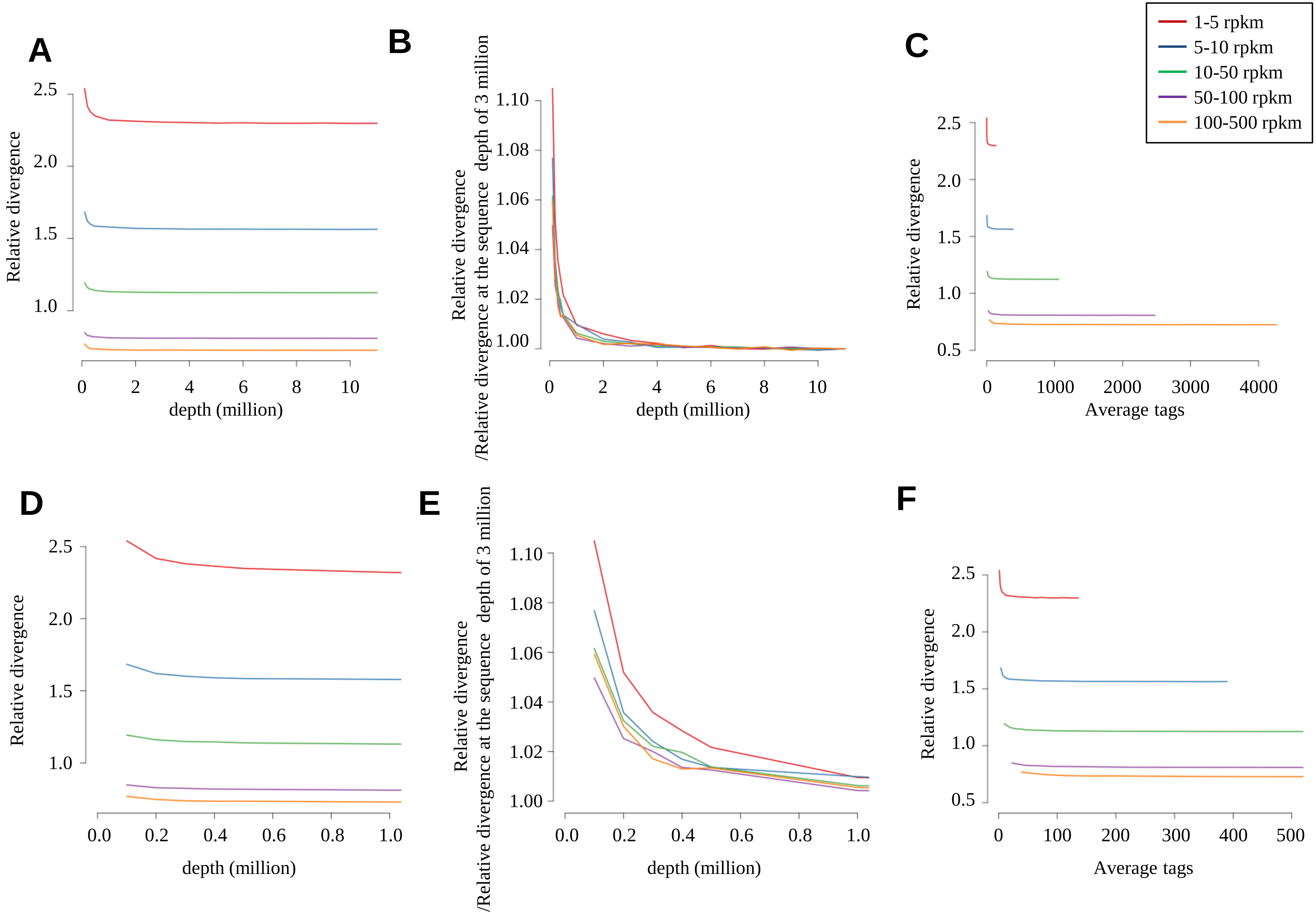
A
Relative divergence
depth (million)
B
Relative divergence /Relative divergence at the sequence depth of 3 million
depth (million)
C
Relative divergence
Average tags
1-5 rpkm
5-10 rpkm
10-50 rpkm
50-100 rpkm
100-500 rpkm
D
Relative divergence
depth (million)
E
Relative divergence /Relative divergence at the sequence depth of 3 million
depth (million)
F
Relative divergence
Average tags

Figure S6

Additional file 1

Figure S6 Dependency of the relative divergences on the sequence depth for the gene of varying average expression levels. (**A**-**C**) Dependency of the calculated relative divergence on the varying sequence depth per cell is shown for each of the gene groups of the indicated expression levels. Average values for the indicated populations are shown. (**D**-**F**) Magnification of the upper panels at the region of small values of x-axis. Dependency of the relative divergences (**A**, **C**, **D** and **F**) or relative divergences compared to those calculated using the average sequence depth of 3 million (**B** and **E**) are shown at the indicated sequence depth (**A**, **B**, **D** and **E**) or at the sequence depth where the indicated number of the sequence tags (**C** and **F**) are corresponded to the indicated gene group.

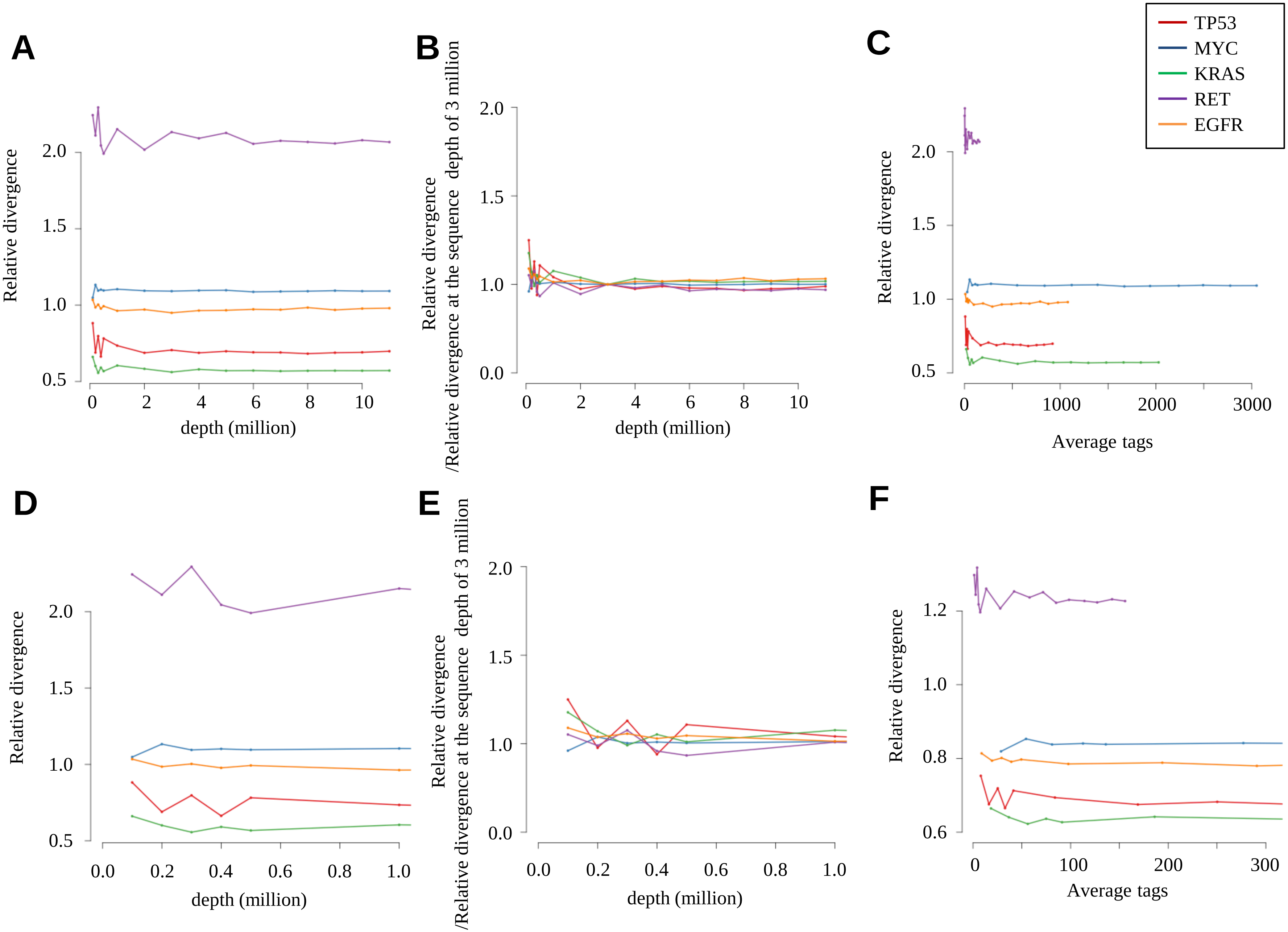

A
B
C
D
E
F
TP53
MYC
KRAS
RET
EGFR
Relative divergence
depth (million)
Relative divergence
/Relative divergence at the sequence depth of 3 million
Average tags
0.5
1.0
1.5
2.0
0.0
0
2
4
6
8
10
1000
2000
3000
0.2
0.4
0.6
0.8
1.2
100
200
300

Figure S7

Additional file 1

Figure S7 Dependency of the relative divergences on the sequence depth for the cancer-related genes. Legends for the graphs are as shown in Additional file 1: Figure S6.

A

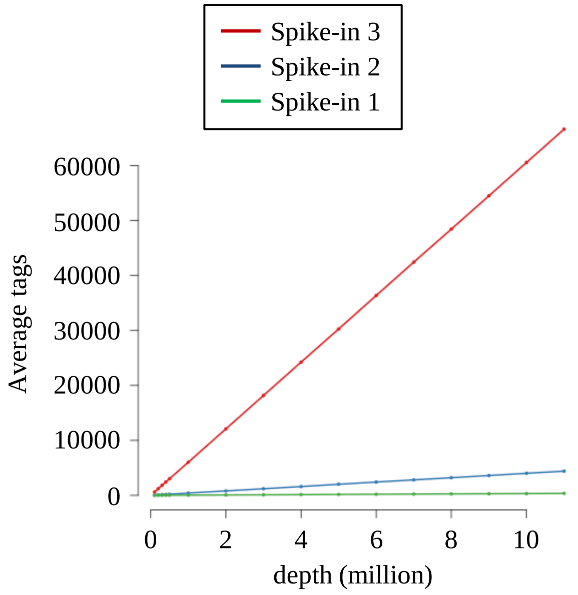

B

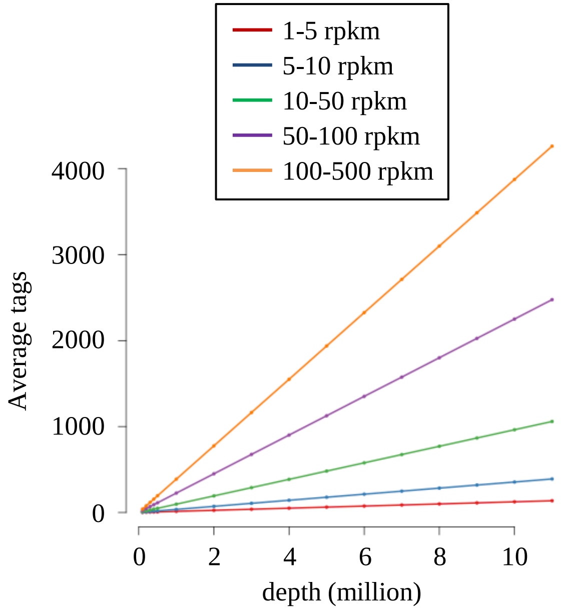

Range	Num of genes
1 ≤ rpkm < 5	2,370
5 ≤ rpkm < 10	1,014
10 ≤ rpkm < 50	3,489
50 ≤ rpkm < 100	541
100 ≤ rpkm < 500	429

C

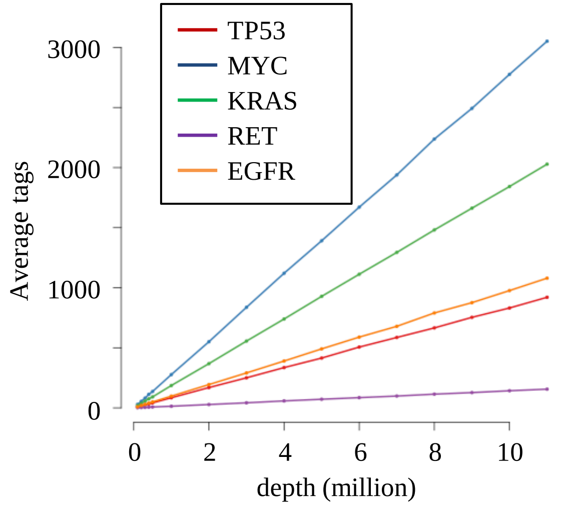

Figure S8

Additional file 1

Figure S8 Relations between the sequence depths and the number of tags in respective genes. Average numbers of tags in spike-in controls (**A**), the gene groups of the indicated expression levels (**B**) and the cancer-related genes (**C**) were shown at the indicated sequence depth.

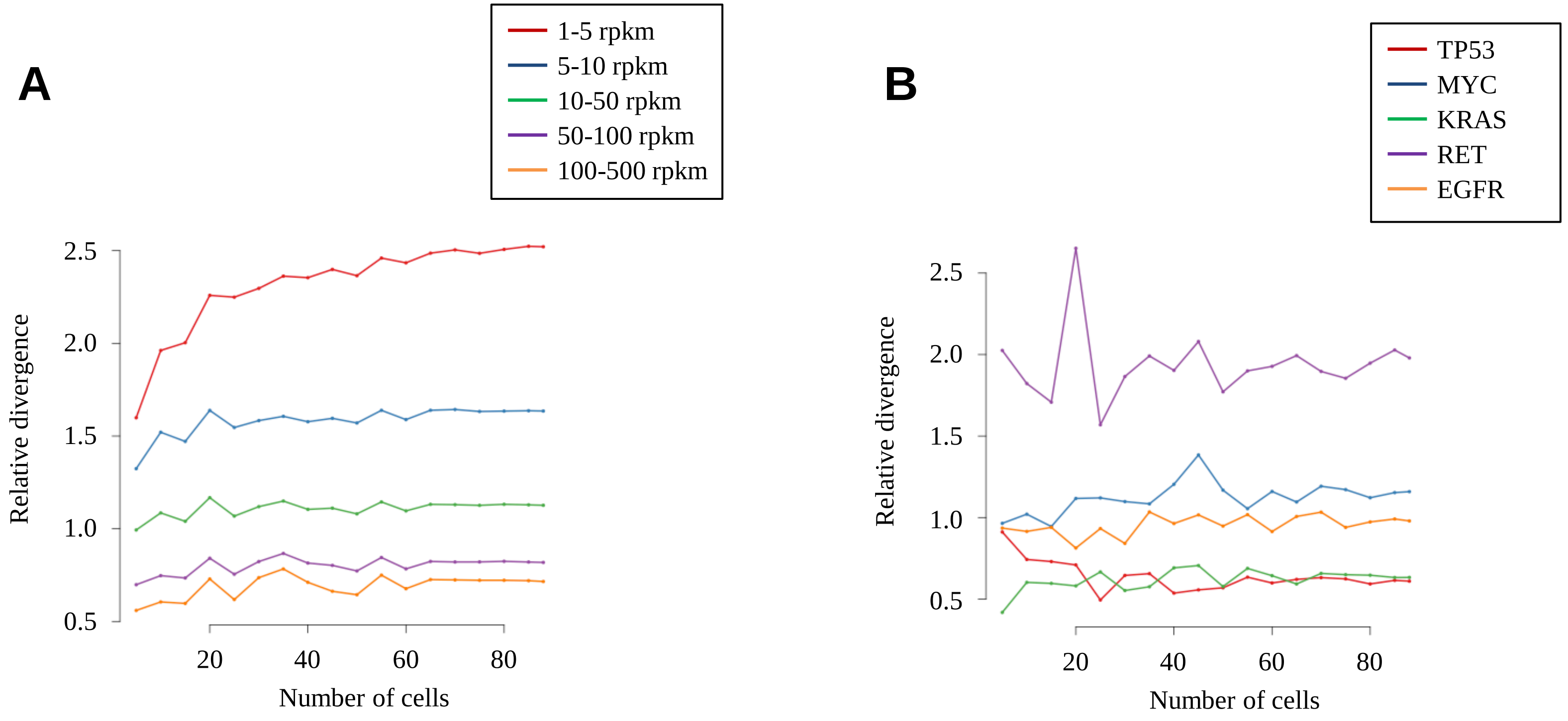
A
1-5 rpkm
5-10 rpkm
10-50 rpkm
50-100 rpkm
100-500 rpkm
Relative divergence
2.5
2.0
1.5
1.0
0.5
20
40
60
80
Number of cells
B
TP53
MYC
KRAS
RET
EGFR
Relative divergence
2.5
2.0
1.5
1.0
0.5
20
40
60
80
Number of cells

Figure S9

Additional file 1

Figure S9 (**A**) Dependency of the calculated relative divergence on the varying number of the cells used for the analysis is shown for each of the gene groups of the indicated expression levels. Average values for the indicated populations are shown. (**B**) Results of the similar analysis as (**A**) for the genes indicated in the margin. For the analyses, a total of 88 cells from the dataset of LC2/ad (43 cells) + LC2/ad replicate (45 cells) were used.

A

Cell lines	Mapped sequences (R1 + R2)	Mean depth	Coverage (x5)	Num of somatic mutations All	Num of somatic mutations CDS*
LC2/ad	1,265,338,449	41.2	0.91	113,724	456
LC2/ad-R	941,119,422	30.5	0.88	96,136	409
PC-9	1,235,410,075	40.2	0.91	118,571	372
VMRC-LCD	1,270,060,339	41.3	0.91	216,282	847

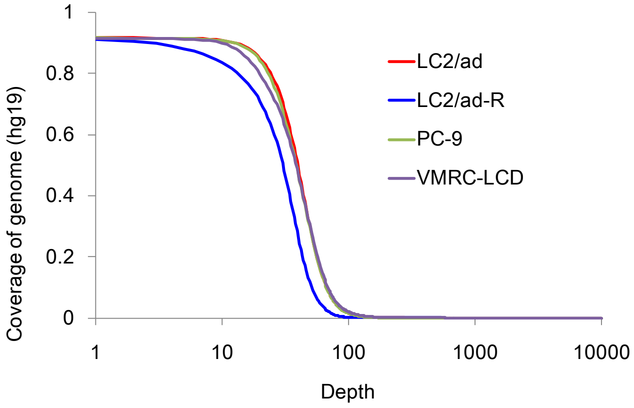

B RET chromosome rearrangement

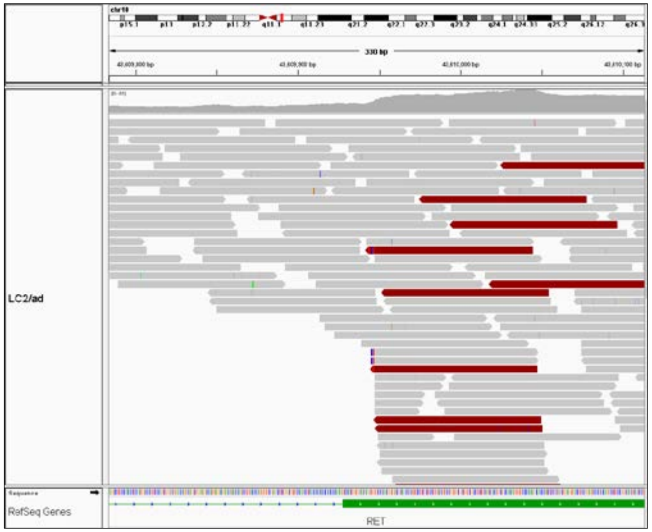

C EGFR E746_A750del

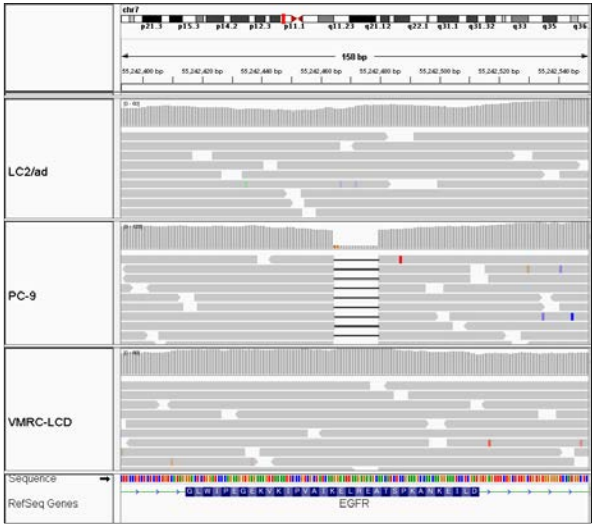

D KRAS G12/13

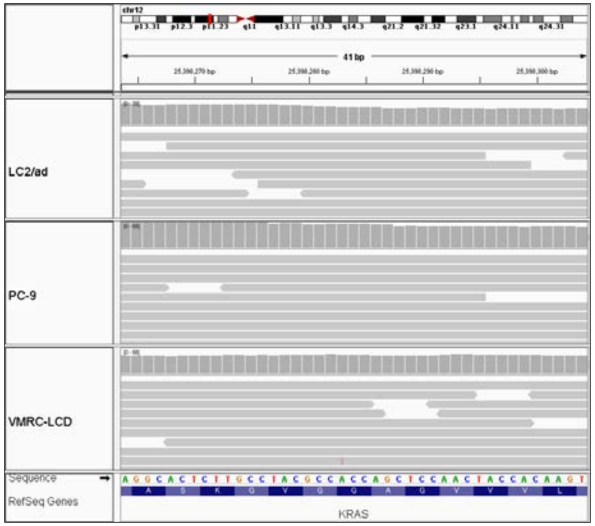

E Number of mutations (LC2/ad vs. LC2/ad-R)

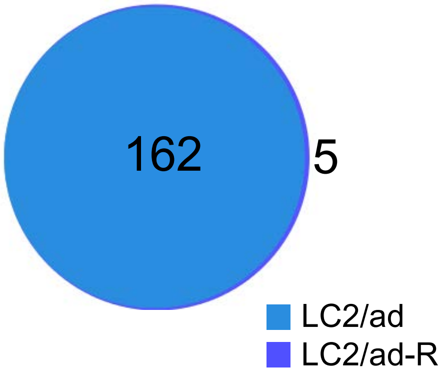

Figure S10

Additional file 1

Figure S10 Information on whole-genome sequences of three cell lines. (**A**) The statistics of whole-genome sequencing data for LC2/ad, LC2/ad-R, PC-9 and VMRC-LCD cells. (**B**) Using whole-genome sequences, chromosome rearrangement of RET in LC2/ad was visualized by IGV. (**C**) (**D**) Well-known driver mutations in lung adenocarcinoma. (**C**) PC-9 harbors E746_A750del mutation in EGFR genes. (**D**) Three cell lines have no KRAS G12 or G13 mutations. (**E**) Comparison of genomic mutations between LC2/ad and LC2/ad-R cells. 167 mutations in CDS were selected; nonsense and stop codon read-through SNVs, missense SNVs with high Polyphen-2 score (probably damaging) and indels, which were supported by sufficient sequence tags (>10 tags) and not reported as germline variations in public databases. 162 mutations were detected in both LC2/ad and LC2/ad-R cells by manual inspection (≥2 variant tags visualized by IGV). Five SNVs were detected as LC2/ad-R-specific mutations. To our knowledge, these five mutations were not reported as driver mutations in previous studies.

A

LC2/ad (replicate)

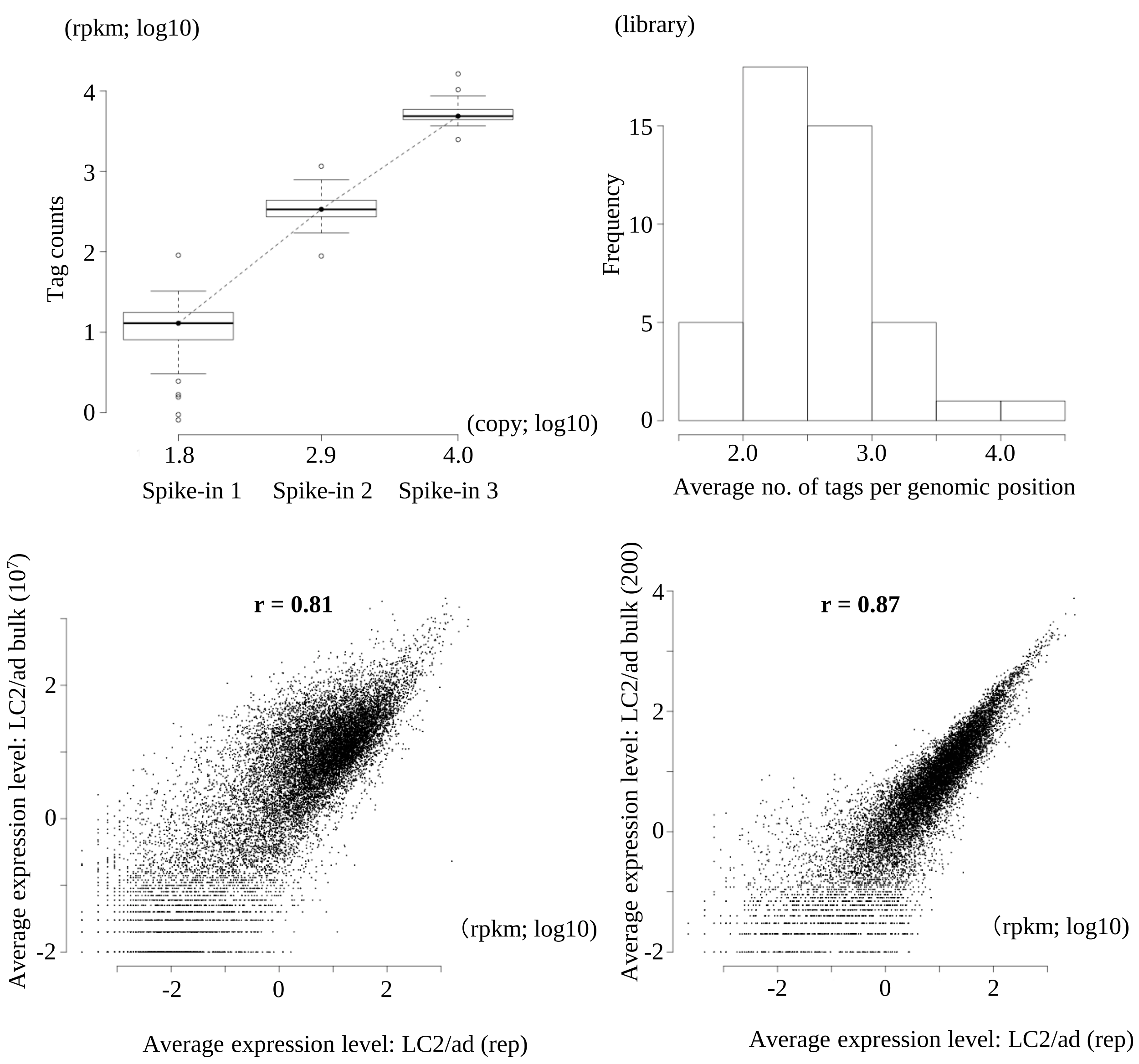

Figure S11A

B

PC-9

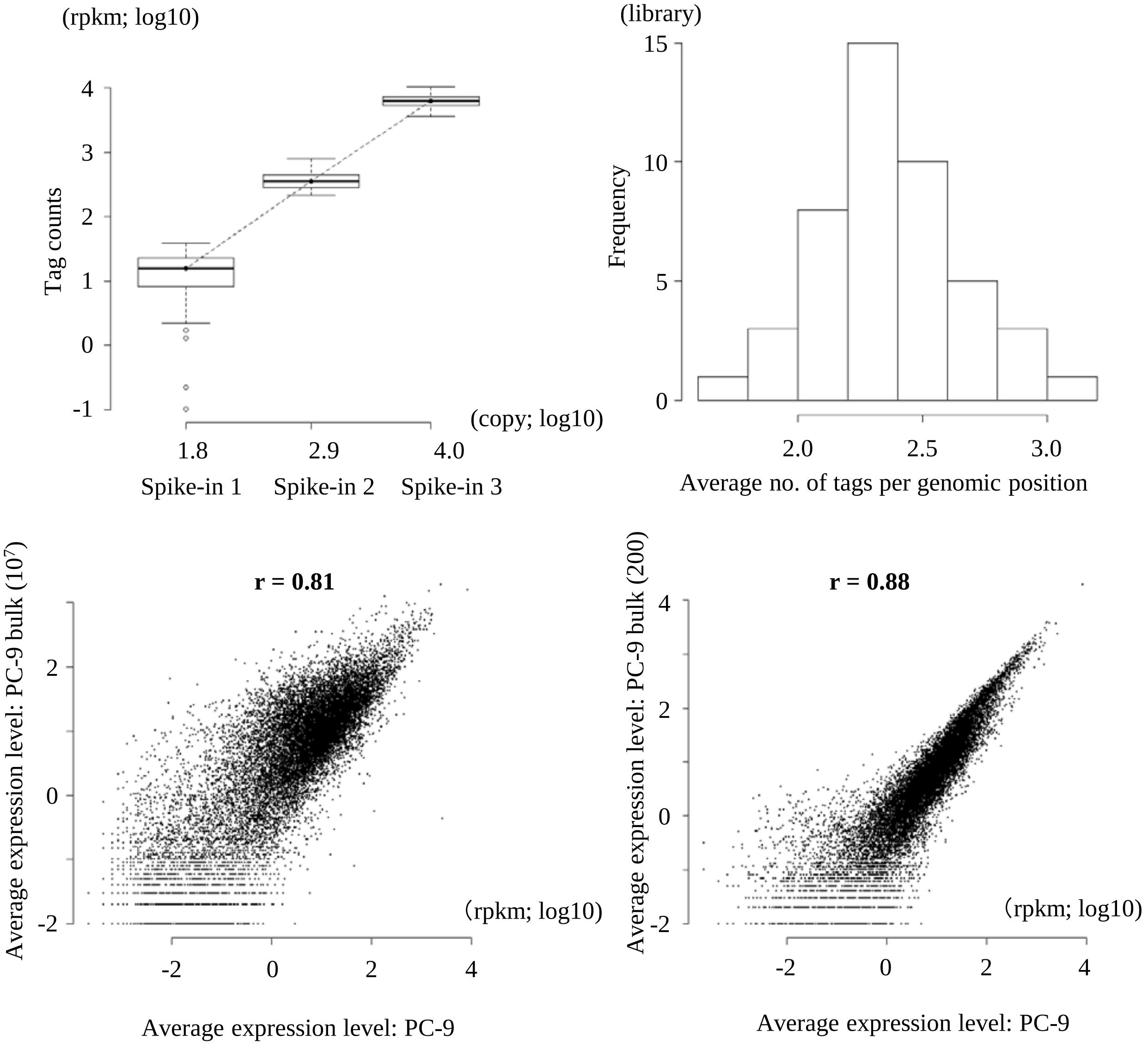

Figure S11B

C

VMRC-LCD

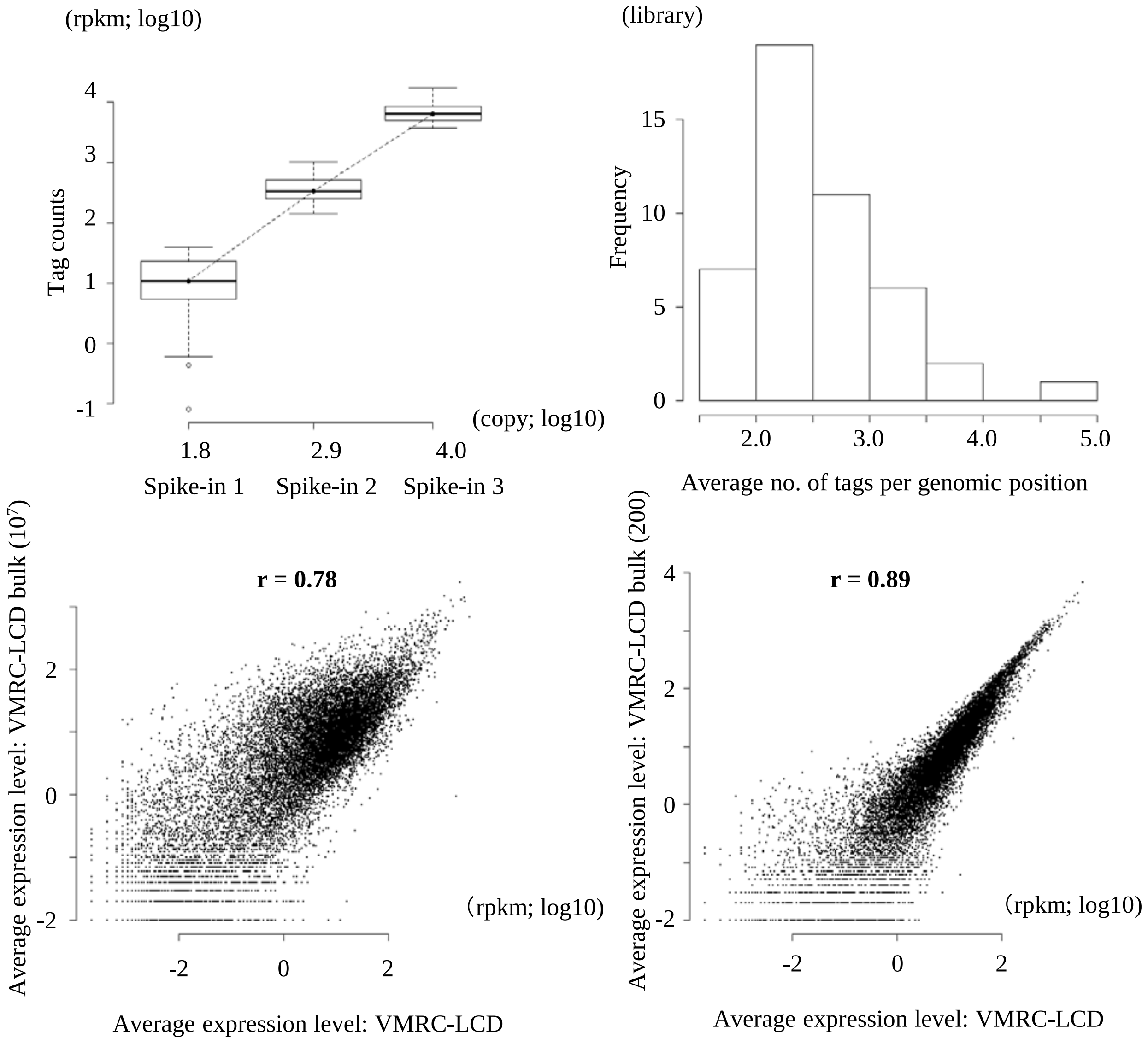

Figure S11C

D

LC2/ad-R

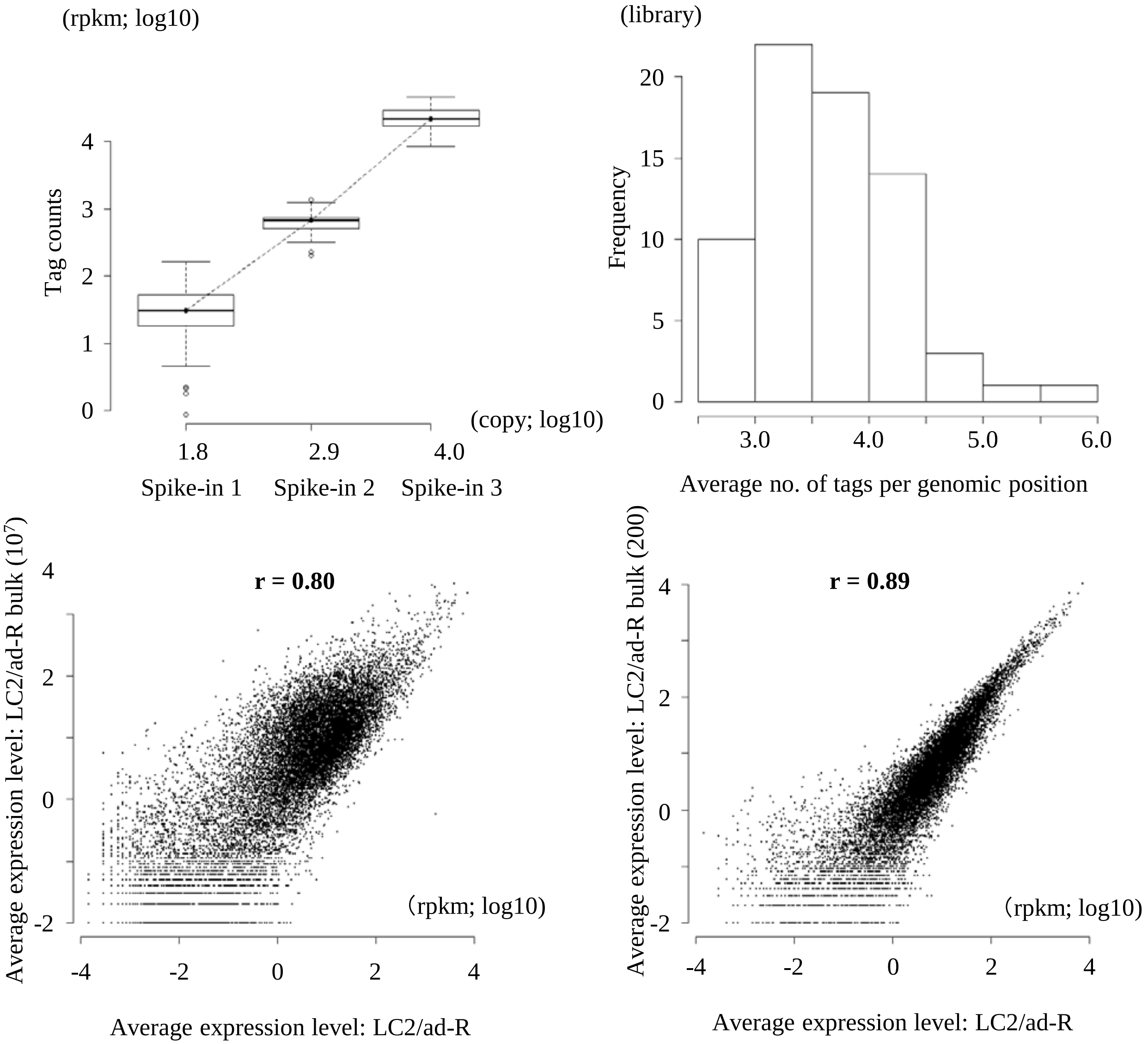

Figure S11D

Additional file 1

Figure S11 RNA-Seq tags generated from different cell lines. Results of the same analysis as shown in Figure 1 for LC2/ad (biological replicate; **A**), PC-9 (**B**), VMRC-LCD (**C**) and LC2/ad-R (**D**) cells. Legends for the figures are as shown in the legend for Figure 1.

MYC amplification in LC2/ad

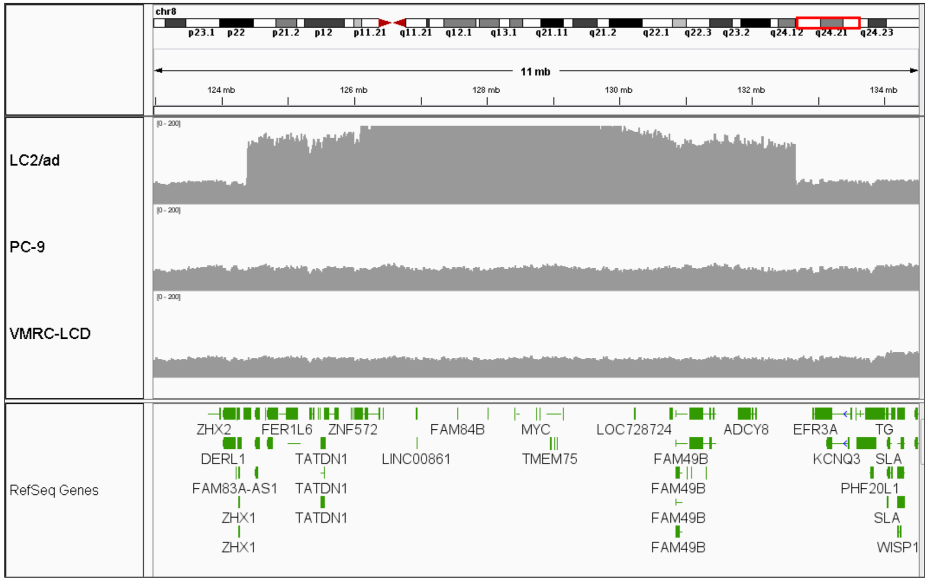

CCNC amplification in VMRC-LCD

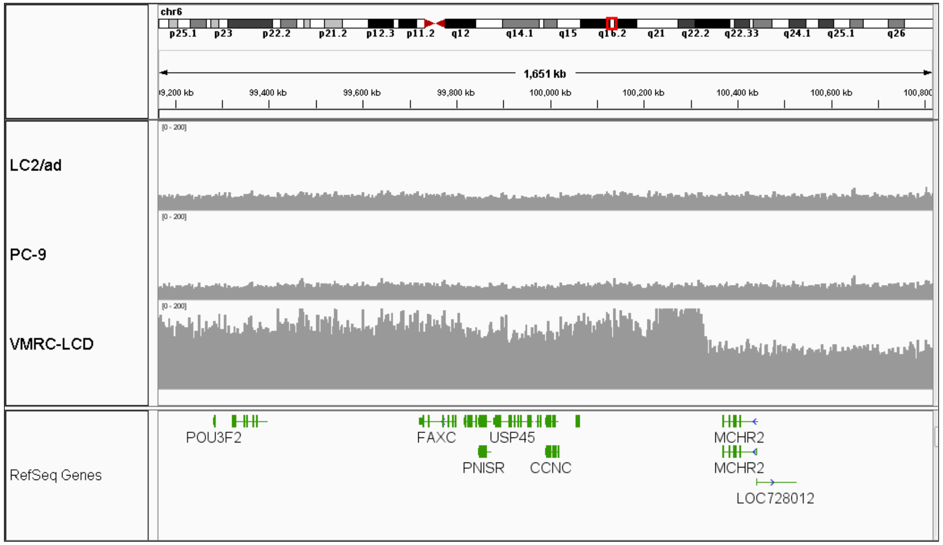

Figure S12

Additional file 1

Figure S12 Amplifications detected by whole-genome sequences. Using whole-genome sequencing data, genomic amplification was detected and visualized by IGV. LC2/ad has amplification around the MYC gene (upper panel) and VMRC-LCD has amplification in the CCNC gene region (lower panel).

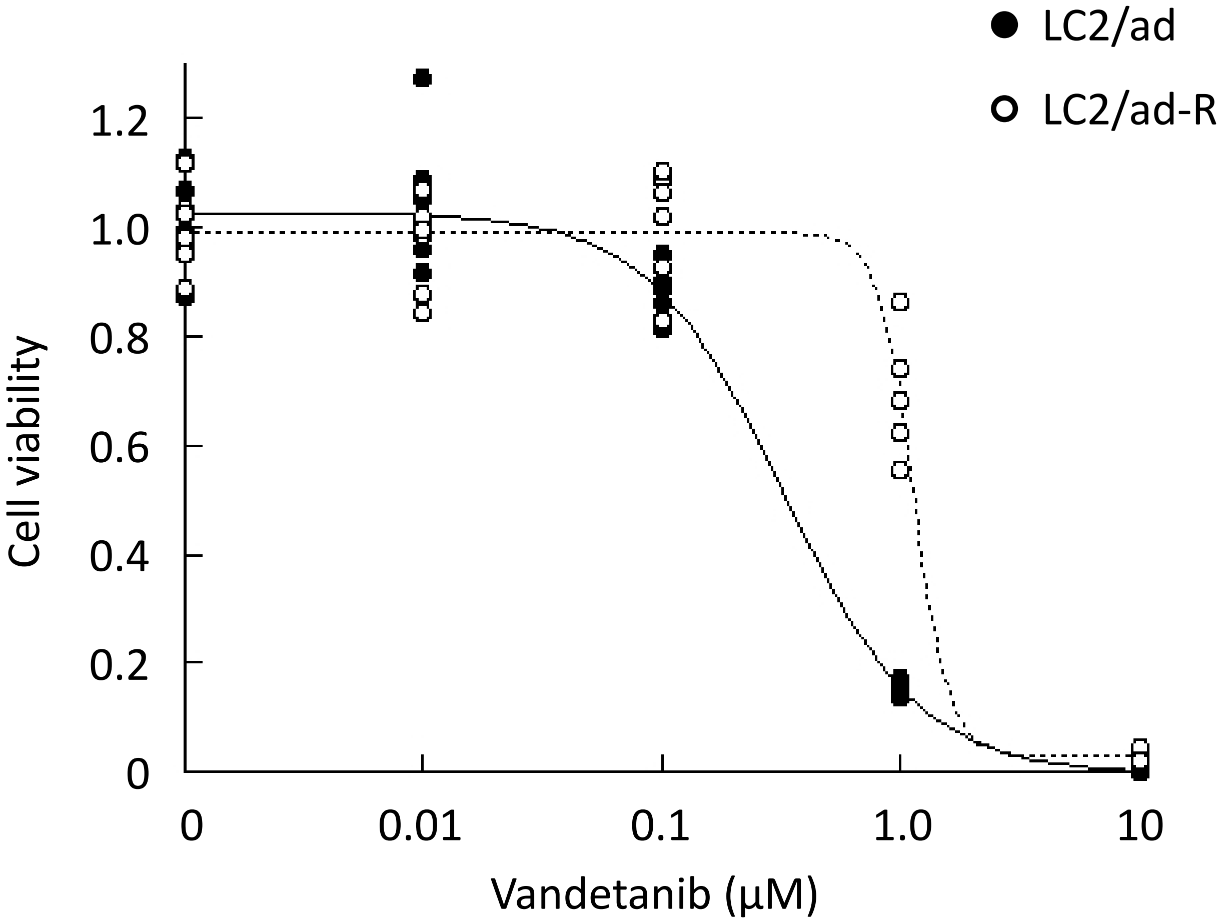
LC2/ad
LC2/ad-R
Cell viability
1.2
1.0
0.8
0.6
0.4
0.2
0
0
0.01
0.1
1.0
10
Vandetanib (μM)

Figure S13

Additional file 1

Figure S13 Drug response of LC2/ad and LC2/ad-R cells. Cell viability at the indicated concentration of vandetanib is shown for LC2/ad (solid circle) and LC2/ad-R (blank circle) cells. The dotted curve represents the regression curve of the dose dependency of the cellular viability for the indicated cell lines.

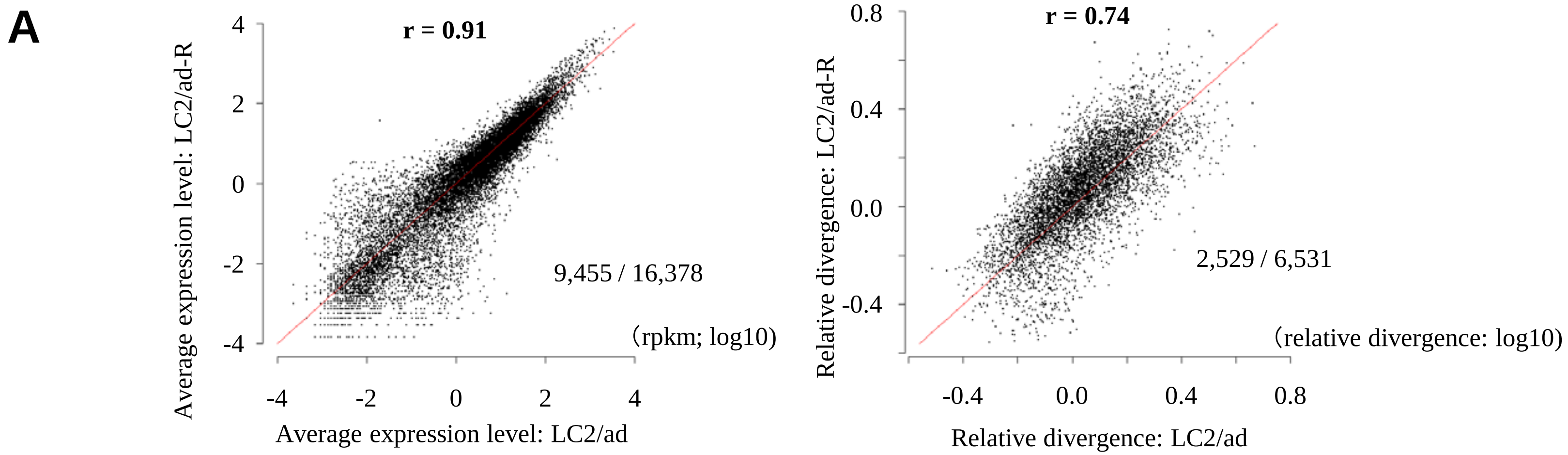
A
r = 0.91
9,455 / 16,378
(rpkm; log10)
Average expression level: LC2/ad-R
Average expression level: LC2/ad
r = 0.74
2,529 / 6,531
(relative divergence: log10)
Relative divergence: LC2/ad-R
Relative divergence: LC2/ad

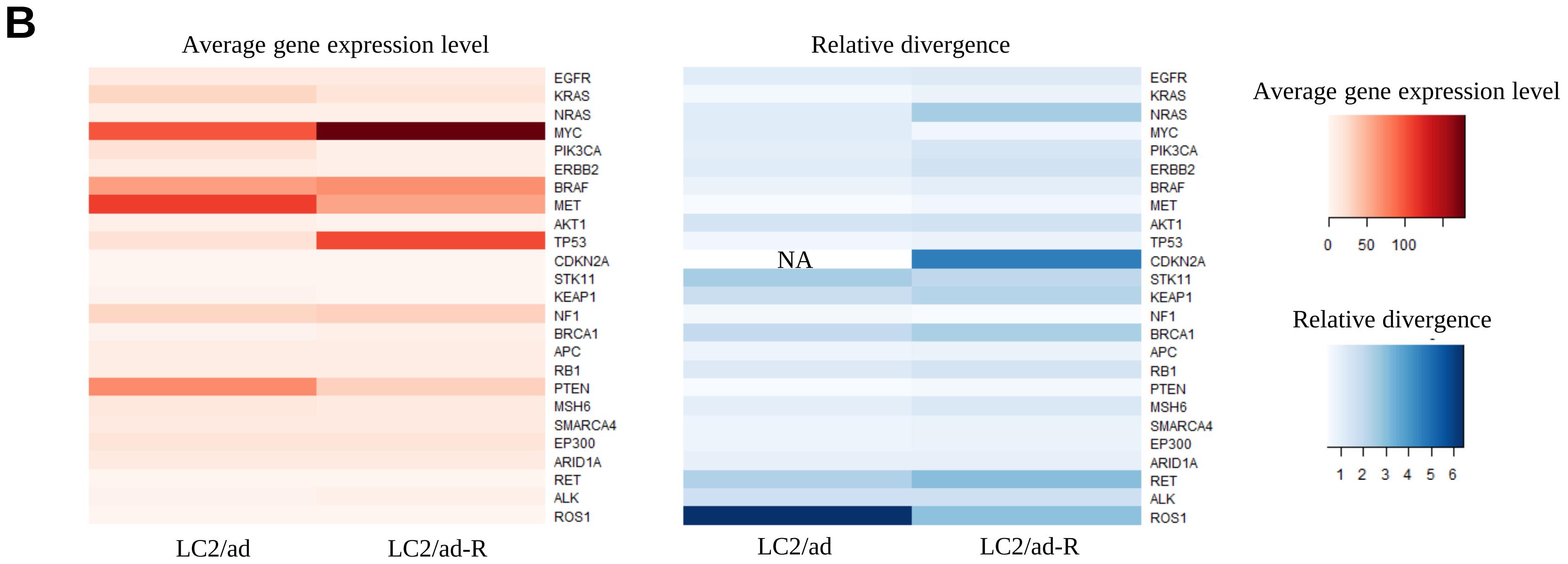
B
Average gene expression level
Relative divergence
EGFR
KRAS
NRAS
MYC
PIK3CA
ERBB2
BRAF
MET
AKT1
TP53
CDKN2A
STK11
KEAP1
NF1
BRCA1
APC
RB1
PTEN
MSH6
SMARCA4
EP300
ARID1A
RET
ALK
ROS1
NA
LC2/ad
LC2/ad-R
Average gene expression level
0 50 100
Relative divergence
1 2 3 4 5 6

Figure S14

Additional file 1

Figure S14 Comparison of the gene expression differences between LC2/ad and LC2/ad-R. (**A**) Comparison of average expression levels (left panel) and the relative divergences (right panel) between LC2/ad and LC2/ad-R cells under an unstimulated condition. Pearson’s correlation coefficients are shown in the plots. (**B**) For the cancer-related genes, heat maps for the average gene expression levels (left) and the relative divergences of the LC2/ad and LC2/ad-R cell lines under an unstimulated condition. The color keys are shown in the margin.

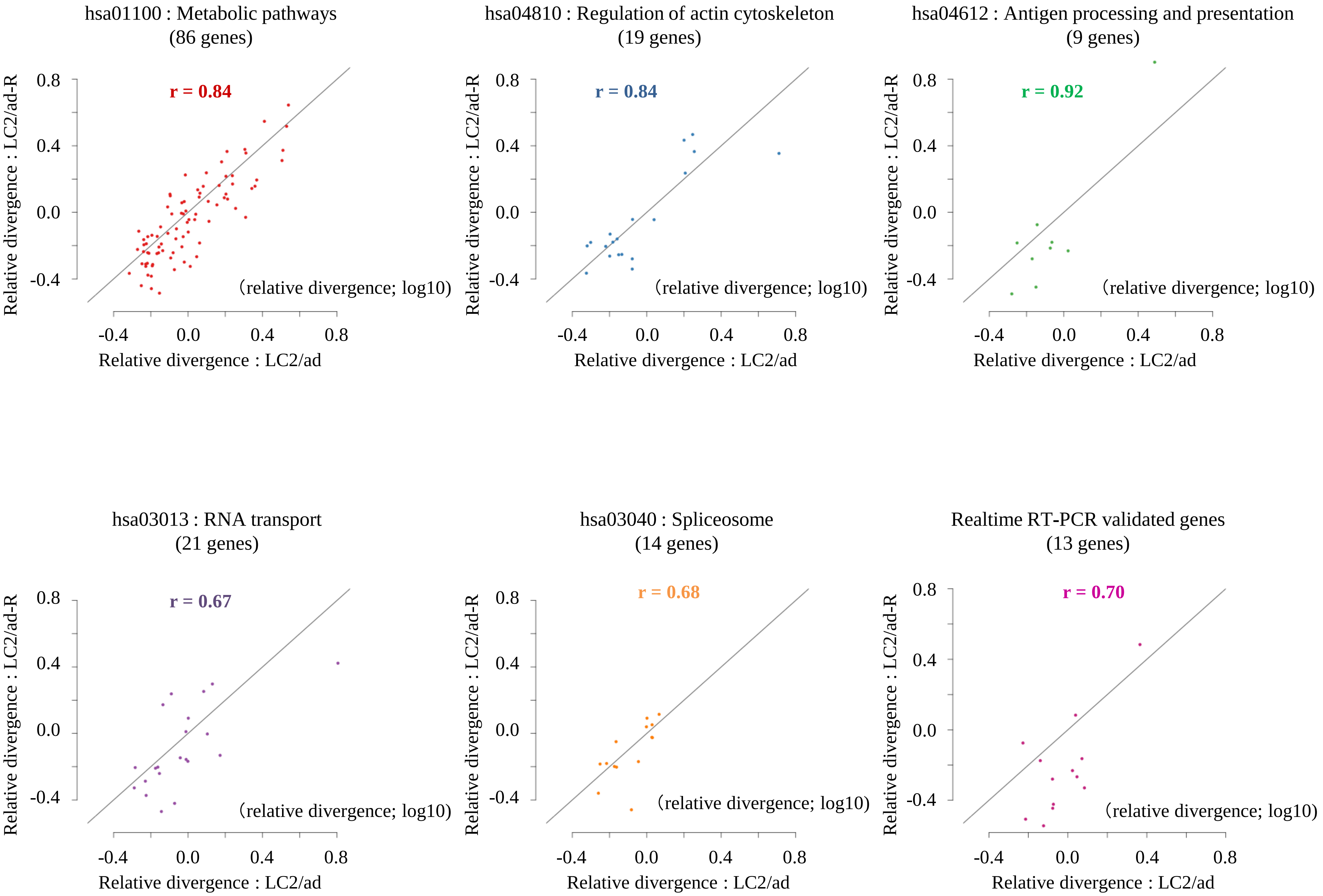
hsa01100 : Metabolic pathways
(86 genes)
r = 0.84
(relative divergence; log10)
hsa04810 : Regulation of actin cytoskeleton
(19 genes)
r = 0.84
(relative divergence; log10)
hsa04612 : Antigen processing and presentation
(9 genes)
r = 0.92
(relative divergence; log10)
hsa03013 : RNA transport
(21 genes)
r = 0.67
(relative divergence; log10)
hsa03040 : Spliceosome
(14 genes)
r = 0.68
(relative divergence; log10)
Realtime RT-PCR validated genes
(13 genes)
r = 0.70
(relative divergence; log10)
Relative divergence : LC2/ad-R
Relative divergence : LC2/ad
-0.4
0.0
0.4
0.8

Figure S15

Additional file 1

Figure S15 Relative divergences of other house-keeping genes in LC2/ad and LC2/ad-R. House-keeping genes belonging to the indicated KEGG categories were selected based on a previous paper and were subjected to the similar analysis as conducted in Figure 5A. Pearson's correlation coefficients are also shown on the plot. Lists of the genes used for the analysis are also shown in Additional file 7: Table S9. Figure S16 Gene expression changes in response to vandetanib. Fold inductions of the average gene expression levels (left panels) and the relative divergences (right panels) in response to vandetanib in LC2/ad and LC2/ad-R. Red line shows the line of no fold induction and dotted blue lines show the line of two-fold (0.5-fold) inductions.

Reference:

Eisenberg E, Levanon EY. Human housekeeping genes are compact. Trends Genet. 2003;19:362–5.

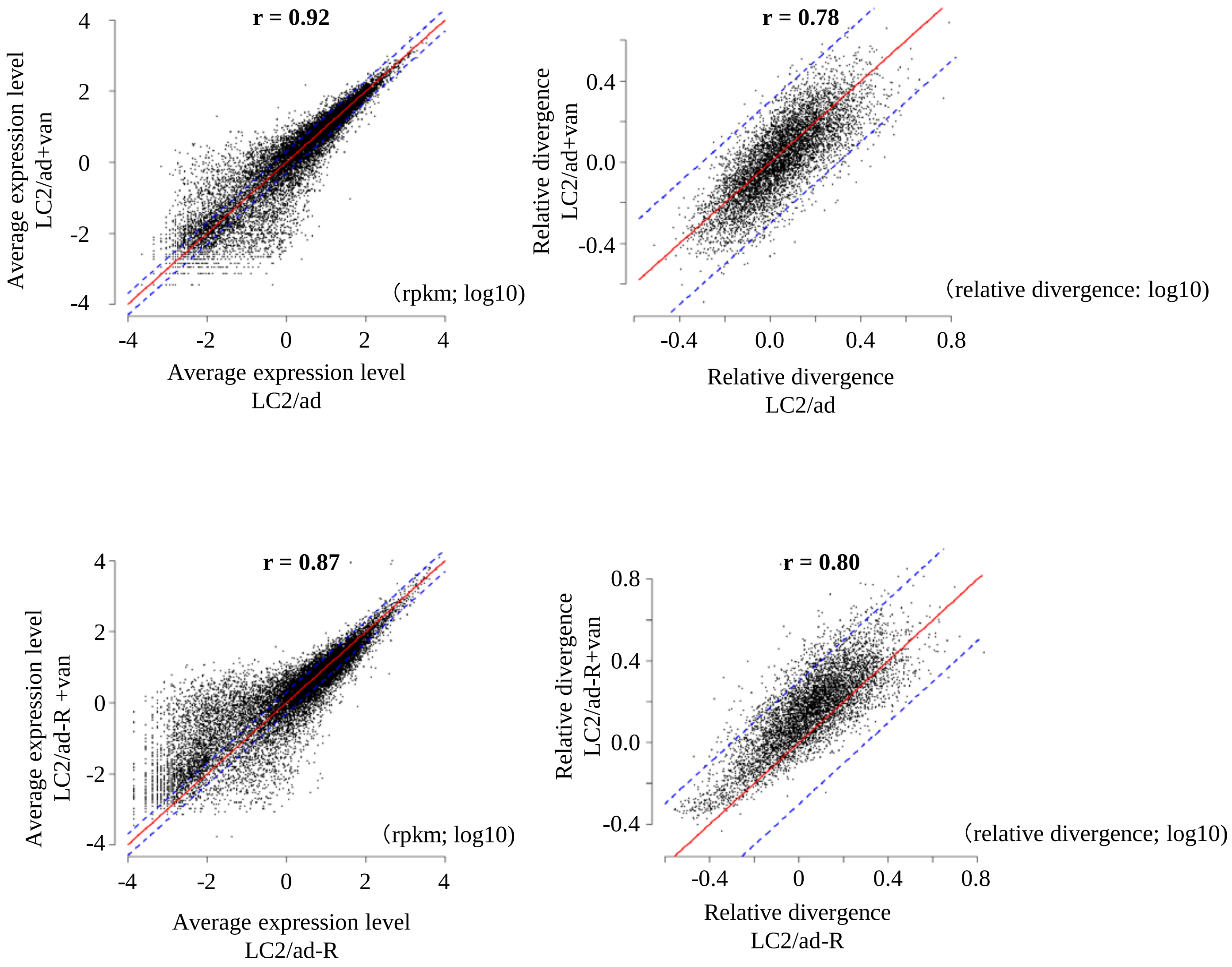
r = 0.92
Average expression level
LC2/ad+van
(rpkm; log10)
Average expression level
LC2/ad
r = 0.78
Relative divergence
LC2/ad+van
(relative divergence: log10)
Relative divergence
LC2/ad
r = 0.87
Average expression level
LC2/ad-R +van
(rpkm; log10)
Average expression level
LC2/ad-R
r = 0.80
Relative divergence
LC2/ad-R+van
(relative divergence; log10)
Relative divergence
LC2/ad-R

Figure S16

Additional file 1

Figure S16 Gene expression changes in response to vandetanib. Fold inductions of the average gene expression levels (left panels) and the relative divergences (right panels) in response to vandetanib in LC2/ad and LC2/ad-R. Red line shows the line of no fold induction and dotted blue lines show the line of two-fold (0.5-fold) inductions***.***

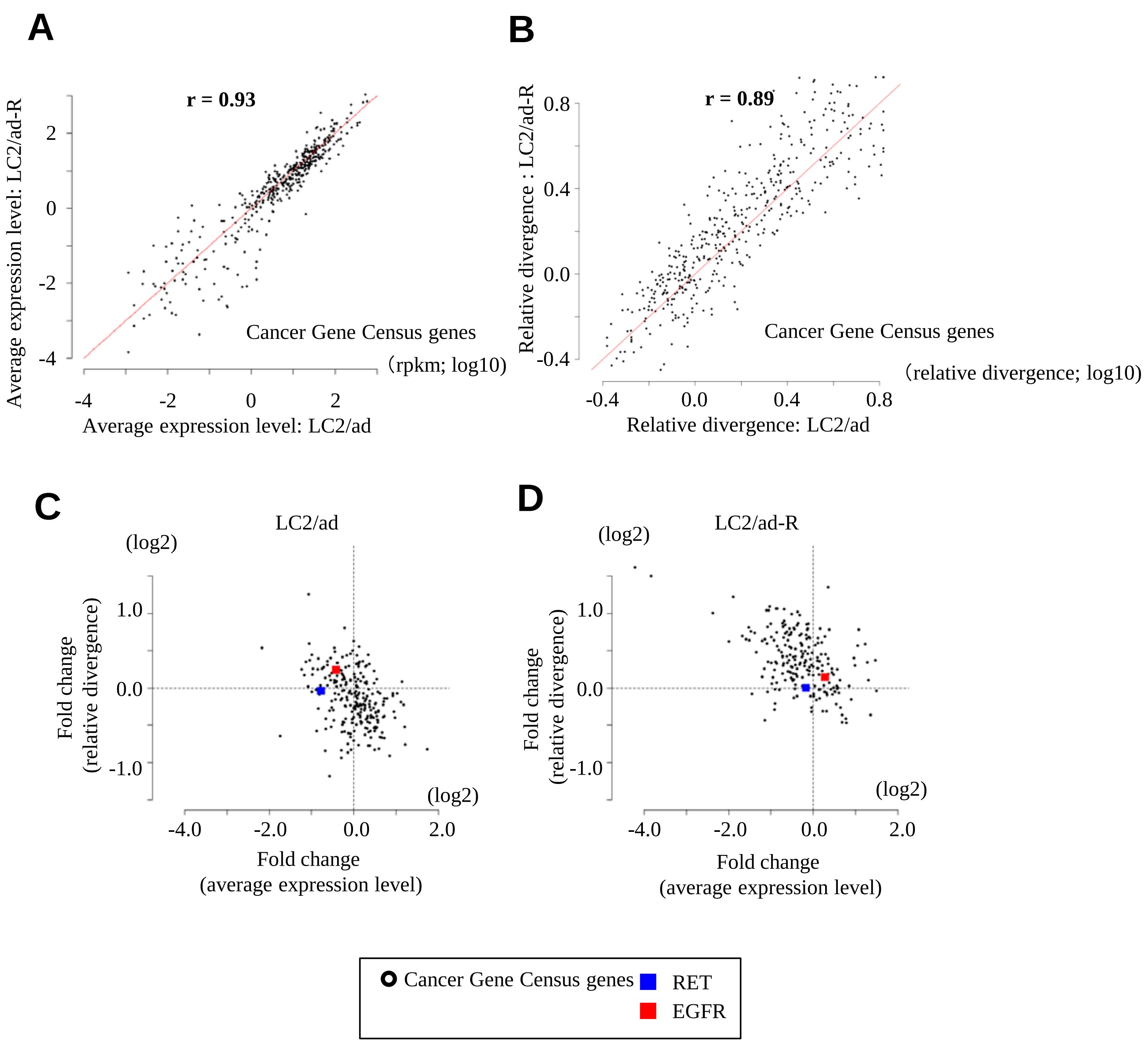
A
r = 0.93
Average expression level: LC2/ad-R
Cancer Gene Census genes
(rpkm; log10)
Average expression level: LC2/ad
B
r = 0.89
Relative divergence : LC2/ad-R
Cancer Gene Census genes
(relative divergence; log10)
Relative divergence: LC2/ad
C
LC2/ad
(log2)
Fold change
(relative divergence)
(log2)
Fold change
(average expression level)
D
LC2/ad-R
(log2)
Fold change
(relative divergence)
(log2)
Fold change
(average expression level)
Cancer Gene Census genes
RET
EGFR

Figure S17

Additional file 1

Figure S17 Gene expression changes of Cancer Gene Census genes. (**A**, **B**) Comparison of the average gene expression levels (**A**) and the relative divergences (**B**) for the Cancer Gene Census genes in LC2/ad an LC2/ad-R cells. Pearson's correlation coefficients are shown in the plots. (**C**, **D**) Fold inductions of the average expression levels (x-axis) and the relative divergences (y-axis) for the Cancer Gene Census genes in response to vandetanib in LC2/ad. (**C**) and LC2/ad-R (**D**). Dotted lines represent the lines of no fold inductions (fold = 1). The plots of the EGFR and RET genes are highlighted by blue and red boxes, respectively..

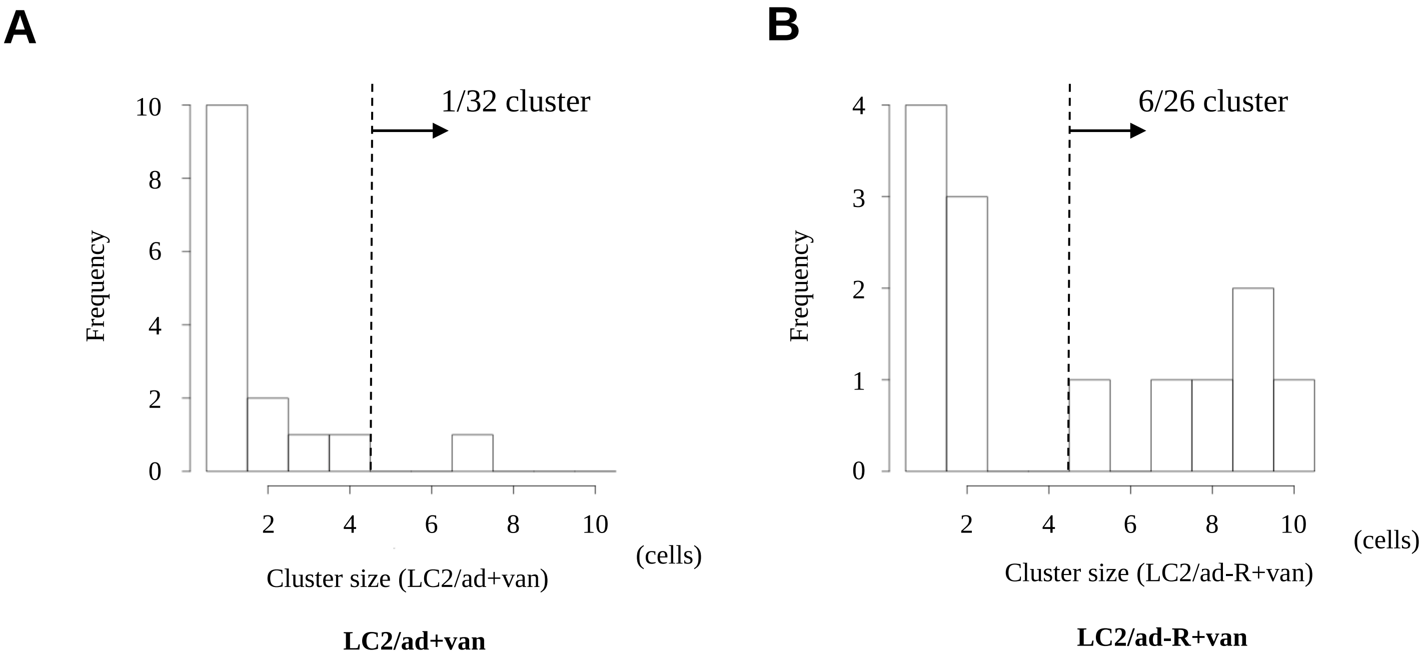
A
1/32 cluster
10
8
6
4
2
0
Frequency
2
4
6
8
10
(cells)
Cluster size (LC2/ad+van)
LC2/ad+van
B
6/26 cluster
4
3
2
1
0
Frequency
2
4
6
8
10
(cells)
Cluster size (LC2/ad-R+van)
LC2/ad-R+van

Figure S18

Additional file 1

Figure S18 Size of the clusters in LC2/ad and LC2/ad-R stimulated with vandetanib. Number of clusters consisting of the indicated number of cells for LC2/ad (**A**) and LC2/ad-R (**B**) stimulated with vandetanib. Number of the clusters belonging to the population indicated by a broken line and an arrow out of the total number of the clusters is shown in the plot. Statistical significance in the difference for the occurrence of the clusters of ≥5 cells out of the total number of clusters (1/32 versus 6/26) was $p = 0.05$ (Fisher's exact test).

		in RefSeq (%)	Average Complexity	Number of genes (rpkm ≥5.0)
bulk	LC2/ad	66,744,270 (86%)	1.58	9,106
	LC2/ad-R	66,322,373 (91%)	1.85	8,459
	PC-9	52,573,421 (87%)	1.58	9,423
	VMRC-LCD	63,516,303 (76%)	1.48	8,611
single cell	LC2/ad	3,581,044 (78%)	2.26	7,495
	LC2/ad-R	7,052,916 (75%)	3.66	7,115
	PC-9	5,726,548 (77%)	2.37	7,769
	VMRC-LCD	5,059,440 (74%)	2.53	7,321

Table S2

Additional file 1

Table S2 Comparison of RNA-Seq statistics between bulk and single-cell libraries.

Gene	Description	Fwd	Rev	Product length
RPS18	Ribosomal protein	GAGGATGAGGTGGAACGTGT	GGACCTGGCTGTATTTTCCA	115
RPL5	Ribosomal protein	CAAGTGGAGGTGACTGGTGA	CCTGCATCCAAATAGCAGGT	83
ATP5F1	House-keeping gene	AGGCTTCCATCCAACACATC	AAAGGTAATGGCGCTTCTGA	81
ACTB	House-keeping gene	GATGAGATTGGCATGGCTTT	CACCTTCACCGTTCCAGTTT	100
GAPDH	House-keeping gene	CGACCACTTTGTCAAGCTCA	GGTGGTCCAGGGGTCTTACT	112
EGFR	Cancer-related gene	TATGTTCCCTCCAGGTCAGC	TGTGGCCAGAGCTGTAAGTG	108
MYC	Cancer-related gene	AGCGACTCTGAGGAGGAACA	CTCTGACCTTTTGCCAGGAG	87
RET	Cancer-related gene	ACAGGGGATGCAGTATCTGG	AAGCCGAAATCCGAAATCTT	108
KRAS	Cancer-related gene	CACGGTCATCCAGTGTTGTC	TTGATTTGTCAGCAGGACCA	115
B2M	House-keeping gene	GAATTCACCCCCACTGAAAA	CCTCCATGATGCTGCTTACA	111
RPL8	Ribosomal protein	CCGAATTGACAAACCCATCT	GGATGCTCCACAGGATTCAT	111
RPS16	Ribosomal protein	GTCACGTGGCCCAGATTTAT	TCTCCTTCTTGGAAGCCTCA	191
RPL37A	Ribosomal protein	GATCTGGCACTGTGGTTCCT	TTCTGATGGCGGACTTTACC	95

Table S4

Additional file 1

Table S4 Primer sequences for real time RT-PCR assays of 13 genes.

PC-9

KEGG ID	description	Total No. of genes	No. of diverse genes	Enrichment (p-value; Fisher’s test)
hsa04110	Cell cycle	124	7	6e-4
hsa04115	p53 signaling pathway	68	4	8e-3
hsa04914	Progesterone-mediated oocyte maturation	86	4	2e-2

KEGG ID	description	Total No. of genes	No. of less-diverse genes	Enrichment (p-value; Fisher’s test)
hsa03010	Ribosome	89	11	2e-5
hsa04520	Adherens junction	73	6	1e-2
hsa05213	Endometrial cancer	52	4	4e-2

VMRC-LCD

KEGG ID	description	Total No. of genes	No. of diverse genes	Enrichment (p-value; Fisher’s test)
hsa04974	Protein digestion and absorption	81	5	4e-3
hsa05322	Systemic lupus erythematosus	133	5	3e-2

KEGG ID	description	Total No. of genes	No. of less-diverse genes	Enrichment (p-value; Fisher’s test)
hsa03010	Ribosome	89	13	3e-6
hsa04730	Long-term depression	70	7	4e-3
hsa04664	Fc epsilon RI signaling pathway	79	7	7e-3

Table S6

Additional file 1

Table S6 GO terms and KEGG pathway in which gene expression divergences were the remarkable in PC-9 and VMRC-LCD. For the selection procedure, see the main text (Figure 2H and Materials and Methods).

A

LC2/ad

(+vandetanib; fold change ≥2 or ≤0.5; average expression level)

GO ID	GO term	No. genes	No. enriched genes	p-value
GO0000278	mitotic cell cycle	319	62	2.2e-16
GO0051301	cell division	293	51	5.3e-12
GO0000087	M phase of mitotic cell cycle	92	25	3.4e-11
GO0000236	mitotic prometaphase	84	23	1.5e-10

KEGG ID	description	No. genes	No. enriched genes	p-value
hsa03030	DNA replication	36	15	9.8e-11
hsa00240	Pyrimidine metabolism	100	18	1.1e-5
hsa04115	p53 signaling pathway	68	14	1.4e-5
hsa00520	Amino sugar and nucleotide sugar metabolism	48	10	1.4e-4
hsa00010	Glycolysis / Gluconeogenesis	66	12	1.8e-4
hsa05219	Bladder cancer	42	9	1.9e-4
hsa00563	Glycosylphosphatidylinositol(GPI)-anchor biosynthesis	25	6	6.2e-4

Table S10A

B

LC2/ad-R
(+vandetanib; fold change ≥2 or ≤0.5; average expression level)

GO ID	GO term	No. genes	No. enriched genes	p-value
GO0000278	mitotic cell cycle	319	86	< 1e-100
GO0000398	nuclear mRNA splicing, via spliceosome	167	67	< 1e-100
GO0008380	RNA splicing	230	70	< 1e-100
GO0051301	cell division	293	82	< 1e-100
GO0000087	M phase of mitotic cell cycle	92	37	1.6e-14
GO0016607	nuclear speck	140	47	3.3e-14
GO0000236	mitotic prometaphase	84	34	1.3e-13
GO0071013	catalytic step 2 spliceosome	80	32	9.1e-13
GO0007067	mitosis	183	53	9.3e-13
GO0006396	RNA processing	68	27	5.1e-11
GO0000922	spindle pole	77	29	5.5e-11
GO0005681	spliceosomal complex	77	28	3.0e-10
GO0006397	mRNA processing	190	49	5.9e-10

KEGG ID	description	No. genes	No. enriched genes	p-value
hsa03008	Ribosome biogenesis in eukaryotes	75	26	3.9e-9
hsa03030	DNA replication	36	16	2.7e-8
hsa03015	mRNA surveillance pathway	83	26	4.7e-8
hsa00240	Pyrimidine metabolism	100	24	2.9e-5
hsa03018	RNA degradation	71	18	9.6e-5
hsa03430	Mismatch repair	23	8	3.2e-4

Table S10B

C

LC2/ad
(+vandetanib; fold change ≥2 or ≤0.5; relative divergence)

GO ID	GO term	No. genes	No. enriched genes	p-value
GO0007268	synaptic transmission	374	44	1.3e-8
GO0030054	cell junction	322	39	3.5e-8

KEGG ID	description	No. genes	No. enriched genes	p-value
hsa03010	Ribosome	89	16	9.3e-7
hsa00830	Retinol metabolism	64	13	1.6e-6
hsa00982	Drug metabolism - cytochrome P450	73	12	3.8e-5
hsa00140	Steroid hormone biosynthesis	56	10	5.9e-5
hsa00980	Metabolism of xenobiotics by cytochrome P450	71	11	1.3e-4
hsa04976	Bile secretion	71	11	1.3e-4
hsa00591	Linoleic acid metabolism	30	6	4.4e-4

Table S10C

D

LC2/ad-R
(+vandetanib; fold change ≥2 or ≤0.5; relative divergence)

GO ID	GO term	No. genes	No. enriched genes	p-value
GO0051301	cell division	293	42	1.8e-5
GO0000278	mitotic cell cycle	319	44	3.3e-5
GO0000075	cell cycle checkpoint	130	22	9.8e-5

KEGG ID	description	No. genes	No. enriched genes	p-value
hsa04115	p53 signaling pathway	68	14	1.3e-4
hsa05412	Arrhythmogenic right ventricular cardiomyopathy (ARVC)	74	12	3.3e-3
hsa02010	ABC transporters	44	8	4.8e-3

Table S10D

Additional file 1

Table S10 GO terms and KEGG pathways which are enriched in the genes that showed the fold inductions of ≥ 2 or ≤ 0.5 regarding (**A**, **B**) average gene expression levels and (**C**, **D**) the relative divergences in response to vandetanib.
